# P23H rhodopsin accumulation causes transient disruptions to synaptic protein levels in rod photoreceptors in a model of retinitis pigmentosa

**DOI:** 10.1242/dmm.052256

**Published:** 2025-06-23

**Authors:** Samantha L. Thompson, Sophie M. Crowder, Maryam Hekmatara, Emily R. Sechrest, Wen-Tao Deng, Michael A. Robichaux

**Affiliations:** ^1^ Department of Ophthalmology and Visual Sciences, West Virginia University, Morgantown, WV 26506, USA; ^2^ Department of Biochemistry and Molecular Medicine, West Virginia University, Morgantown, WV 26506, USA

**Keywords:** Rhodopsin, Photoreceptors, Mislocalization, Synapse, Retina, Super-resolution

## Abstract

Rod photoreceptor neurons in the retina detect scotopic light through the visual pigment rhodopsin (Rho) in their outer segment (OS). Efficient Rho trafficking to the OS through the inner rod compartments is critical for long-term rod health. However, given the importance of protein trafficking to the OS, little is known about the trafficking of rod synaptic proteins. Furthermore, the subcellular impact of Rho mislocalization on rod synapses (i.e. ‘spherules’) has not been investigated. In this study, we used super-resolution and electron microscopies, along with proteomics, to perform a subcellular analysis of Rho synaptic mislocalization in P23H-Rho-RFP mutant mice. We discovered that mutant P23H-Rho-RFP protein mislocalized in distinct accumulations within the spherule cytoplasm, which we confirmed with adeno-associated virus overexpression. Additionally, we found specific synaptic protein abundance differences in P23H-Rho-RFP mice. Interestingly, in P23H knock-in mice with no RFP tag, we detected no synaptic protein abundance changes. In rd10 mutant rods, Rho mislocalized along the spherule plasma membrane, and there were synaptic protein abundance differences at postnatal day 20. Our findings demonstrate that some rod photoreceptor synaptic proteins are sensitive to Rho mislocalization.

## Teaser

**Summary:** This study examines the impact of rhodopsin mislocalization on rod photoreceptor synaptic structures and synaptic protein levels using P23H-RFP rhodopsin mutant mice and other mouse models of rhodopsin mislocalization.

## INTRODUCTION

In the retina, rod photoreceptor neurons detect dim light through the photoactivation of the rod-specific G-protein coupled receptor rhodopsin (Rho). Rho and other visual proteins are densely loaded into stacked membrane discs in the rod outer segment (OS) compartment, which is joined to the inner segment (IS) by a narrow connecting cilium. Mammalian rods have compartmentalized cell bodies preceding the presynaptic terminals (spherules), which form synapses with downstream retinal neurons (Townes-Anderson et al., 1988). Proper Rho protein trafficking to the OS is essential for long-term rod stability and retinal health (Sung et al., 1994; Lem et al., 1999). Because new OS membrane discs are continuously formed in rods, Rho protein, which is synthesized throughout the cell body and IS, must be constantly fluxed unidirectionally into the OS through various coordinated trafficking mechanisms. Any disruption to the unidirectional flow of Rho into the OS causes Rho mislocalization, which is the typical subcellular outcome of blinding rod diseases caused either by a genetic variant (Guo et al., 2022; Hagstrom et al., 1999) or retinal detachment (Fariss et al., 1997; Fisher et al., 2005). Despite the central role of OS protein trafficking in rods, a cellular trafficking system in rods is required to supply and maintain their presynaptic spherules; however, little is known about these trafficking mechanisms and how they might be affected by Rho mislocalization.

**Fig. 1.**
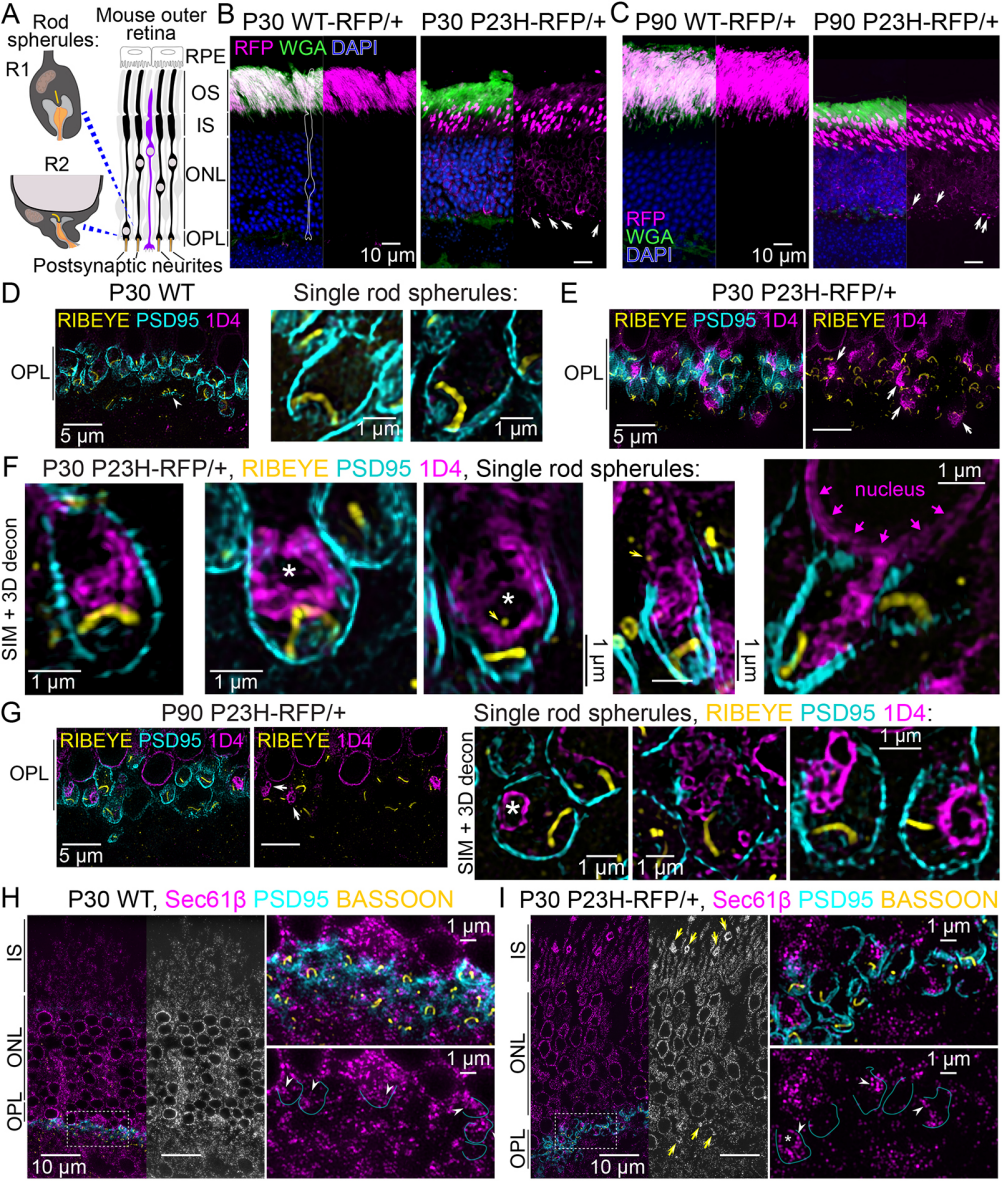
**Mutant P23H-hRho-RFP protein is mislocalized within the cytoplasm of rod photoreceptor presynaptic spherules.** (A) Diagram depicting the layers of the mouse outer retina (RPE, retinal pigment epithelium; OS, outer segment; IS, inner segment; ONL, outer nuclear layer; OPL, outer plexiform layer) and the two types of rod spherules (R1, top; R2, bottom). Spherule illustrations were based on Li et al. (2016) under the terms of the CC-BY 4.0 license. (B,C) Confocal *z*-projections of *WT-RFP/+* and *P23H-RFP/+* retinal cryosections at postnatal day (P)30 (B) and P90 (C). RFP fluorescence is magenta, and sections were co-stained with wheat germ agglutinin (WGA) to label OS membranes (green) and DAPI to label nuclei (blue). White arrows indicate mislocalized RFP. (D) Structured illumination microscopy (SIM) *z*-projections with 3D deconvolution of the OPL from a P30 wild-type (WT) retina. In the images, RIBEYE (yellow)- and PSD95 (cyan)-immunolabeled rod spherules are aligned in the OPL above a cone pedicle (white arrowhead). No 1D4 Rho labeling (magenta) is present in the WT OPL. In single-spherule examples, the RIBEYE^+^ ribbons are horseshoe-shaped structures. (E,F) SIM images of the OPL in a *P23H-RFP/+* retina at P30 with the same immunolabeling as in D. Accumulations of 1D4 immunolabeling in the OPL were localized near the synaptic ribbons (white arrows). (F) Single-spherule SIM examples in *P23H-RFP/+* P30 retinas. 1D4^+^ Rho accumulations are localized in the cytoplasm of the spherules, typically above the ribbon. In the second SIM+3D deconvolution image, a gap in the accumulated 1D4^+^ fluorescence is indicated by a white asterisk. The far-right example is a R2-type mutant spherule with 1D4 fluorescence that surrounds the nucleus (magenta arrows) and extends into the spherule cytoplasm. (G) SIM images of *P23H-RFP/+* retinas at P90 with the same immunolabeling as in D-F. White arrows indicate 1D4^+^ OPL accumulations. In single-spherule examples, cytoplasmic 1D4^+^ accumulated puncta surround gaps in fluorescence (white asterisk). (H,I) SIM *z*-projection images of P30 WT (H) and P30 *P23H-RFP/+* (I) retinas immunolabeled for Sec61β (ER marker, magenta), PSD95 (cyan) and bassoon (Bsn; yellow). In magnified images, the PSD95 rod spherule border is annotated in select rod spherules to demonstrate Sec61β^+^ ER fluorescence within individual WT and *P23H-RFP/+* rod spherules (white arrowheads). In the *P23H-RFP/+* image in I, ER accumulations are labeled with yellow arrows. All experiments were repeated in triplicate (*N*=3 mice per timepoint, per experiment).

Rod presynaptic spherules are located in the outer plexiform layer (OPL) of the retina and contain a tetrad of postsynaptic invaginating neurites (Behrens et al., 2016; Torten et al., 2023). Each rod spherule features a single synaptic ribbon, an electron-dense structure that organizes synaptic vesicles for glutamate release in the dark (Moser et al., 2020). Most mouse rods have stereotypical R1 spherules that are connected to the cell body through an axon [or ‘internal fiber’ (Carter-Dawson and Lavail, 1979)], while fewer rods have R2 spherules that are contiguous with the cell bodies (Fig. 1A; Li et al., 2016). Critically, rod spherules contain essential proteins that are either structural elements of the synaptic ribbon, such as bassoon (Bsn), or proteins that localize at the synaptic cleft to form stabilizing trans-synaptic protein complexes, including ELFN1, dystrophin (Dmd) and dystroglycan (Dag1) (Furukawa et al., 2020). Disruptions to these rod synaptic proteins lead to functional and structural defects in spherules and often cause irreparable synaptic miswiring (Dick et al., 2003; Omori et al., 2012).

Therefore, rods must utilize a secretory system to maintain their presynaptic spherules that operates in concert with constant protein delivery to the OS. In mouse models of retinitis pigmentosa (RP) with photoreceptor degeneration, including for the well-characterized misfolding P23H-Rho mutation (Kaushal and Khorana, 1994; Saliba et al., 2002), Rho is mislocalized not only to the rod IS and cell body but also to the OPL (Barhoum et al., 2008; Hagstrom et al., 1999). In the *P23H-hRho-Tag-RFP-T* mouse, mutant P23H-human Rho (hRho)-RFP protein mislocalized and accumulated throughout the endoplasmic reticulum (ER) in the IS, outer nuclear layer (ONL) and OPL (Robichaux et al., 2022). Rho mislocalization in the OPL has also been demonstrated in RP dog and human retinas (Beltran et al., 2006; Fariss et al., 2000; Milam et al., 1998), as well as after retinal detachment (Fariss et al., 1997; Fisher et al., 2005); however, the impact of Rho OPL mislocalization on rod spherule structure and synaptic protein trafficking and turnover has never been investigated.

Here, we performed a detailed subcellular analysis of Rho mislocalization in rod spherules. Using *P23H-RFP* mice, we found that P23H-hRho-RFP mutant protein accumulated within the spherule cytoplasm, as opposed to membrane mislocalization observed in rd10 RP mutant rods or in overloaded wild-type (WT) rods. Mutant P23H-Rho-RFP accumulation in *P23H-RFP/+* rods interfered with normal rod synaptic protein levels, and we found a decrease in ELFN1 abundance in rd10 rods at postnatal day (P)20. By comparison, synaptic protein levels were not disrupted in P23H-Rho mutant rods with no RFP tag. Thus, our findings indicate that Rho accumulation at rod spherules disrupts normal synaptic protein localization.

## RESULTS

### Mutant P23H-Rho-RFP protein is mislocalized within the cytoplasm of rod presynaptic spherules

To confirm that Rho mislocalization in *P23H-hRho-TagRFP-T* (hereafter *P23H-RFP*) heterozygous knock-in mouse retinas is the outcome of the P23H-Rho misfolding mutation rather than an effect of the C-terminal TagRFP-T fusion tag, a new *WT-hRho-TagRFP-T* mouse line (hereafter *WT-RFP*) was generated with a restored WT P23 residue in the *hRho-RFP-TagRFP-T* knock-in allele. As in the *P23H-RFP* mice, an additional 1D4 sequence is included at the C-terminus of the *WT-RFP* allele. In *WT-RFP/+* heterozygous retinas, WT-hRho-TagRFP-T fusion protein (hereafter WT-hRho-RFP) was localized exclusively in the OS layer (Fig. 1B,C). By comparison, in *P23H-RFP/+* heterozygous mouse retinas, P23H-hRho-TagRFP-T protein (abbreviated as P23H-hRho-RFP) was mislocalized throughout the IS, ONL and OPL at P30 and P90 (Fig. 1B,C), as described previously (Robichaux et al., 2022). The normal OS localization of WT-hRho-RFP demonstrates that Rho mislocalization in *P23H-RFP/+* rods is triggered by the P23H-Rho mutation, not the TagRFP-T fusion tag. Additionally, western blotting with the 1D4 Rho monoclonal antibody (Molday and MacKenzie, 1983) was used to demonstrate a ∼65 kDa band corresponding to the WT-hRho-RFP protein in *WT-RFP/+* retinas (Fig. S1A, magenta arrow). A deglycosylation assay showed a lower molecular mass shift, which confirmed that WT-hRho-RFP was glycosylated like endogenous mouse WT Rho protein and WT Rho-GFP-1D4 protein from the *Rho-GFP-1D4/+* mouse (Haggerty et al., 2024) (Fig. S1B, magenta arrow). ONL density was quantified to compare P30 WT retinas to P30 *WT-RFP/+* retinas, revealing no statistically significant difference (Fig. S1C), which demonstrates that *WT-RFP/+* rods are stable at this age. However, there was ONL photoreceptor nuclei loss in P180 *WT-RFP/+* retinas, indicating that the TagRFP-T tag induces late-stage photoreceptor degeneration, which is a limitation for this model. In a separate analysis, ONL nuclei counts in *WT-RFP/+* retinas were greater than those in age-matched *P23H-RFP/+* retinas at both P30 and P90 (Fig. S1D).

Next, super-resolution structured illumination microscopy (SIM) was used to closely examine the mislocalization of P23H-hRho-RFP mutant protein in the OPL of *P23H-RFP/+* retinas. At P30, *P23H-RFP/+* retinas were shown to be in an early stage of degeneration (Robichaux et al., 2022). Here, P30 *P23H-RFP/+* and age-matched WT retinas were co-immunolabeled with the 1D4 monoclonal Rho antibody, an anti-RIBEYE (also known as CTBP2) antibody to visualize synaptic ribbons and an anti-PSD95 (also known as DLG4) fluorescent nanobody to visualize the presynaptic spherule plasma membranes (Koulen et al., 1998). In the OPL of P30 WT retinas, PSD95^+^ rod spherule plasma membranes encased single RIBEYE^+^ synaptic ribbons that typically appear as horseshoe-shaped structures (Fig. 1D; Fig. S1E). In P30 *P23H-RFP/+* retinas, bright puncta of mislocalized, accumulated Rho protein were located inside some of the rod spherules in the OPL (Fig. 1E, white arrows). Rho puncta were detected in 28.3±8.6% (mean±s.d., *N*=3 mice) of all P30 *P23H-RFP/+* rod spherules imaged with SIM. Upon closer observation of individual *P23H-RFP/+* rod spherules, mutant Rho puncta were localized within the cytoplasm, typically near the synaptic ribbons (Fig. 1F; Fig. S1F,G). In some spherules, there was a partial overlap in 1D4 and RIBEYE signals, and, in some cases, the cytoplasmic P23H-hRho-RFP accumulations were less punctate and instead formed a swirling pattern of bright fluorescence surrounding a dark patch (Fig. 1F, white asterisks). RIBEYE^+^ synaptic ribbons appeared intact in most of the mutant *P23H-RFP/+* spherules examined. We also observed cases of a small RIBEYE^+^ puncta in the cytoplasmic space in both WT and *P23H-RFP/+* spherules; in *P23H-RFP/+* spherules, these RIBEYE^+^ puncta sometimes colocalized with the mutant P23H-hRho-RFP puncta (Fig. 1F; Fig. S1G). These small RIBEYE^+^ puncta could represent recycling synaptic ribbons (Adly et al., 1999; Schmitz et al., 2012). Although most 1D4^+^ P23H-hRho-RFP accumulations were observed as distinct puncta localized near a synaptic ribbon, in some cases, such as in R2 spherules, the 1D4^+^ P23H-hRho-RFP in the spherule cytoplasm formed a continuous network with the cytoplasm surrounding the adjacent cell body nucleus (Fig. 1F, magenta arrows).

The same SIM analysis was performed in *P23H-RFP/+* mice at P90. At this age, *P23H-RFP/+* mice exhibit photoreceptor degeneration, but the surviving rods were shown to be adaptive to the mutation, as the rate of degeneration and cell loss reduces after P90 (Robichaux et al., 2022). Here, in P90 mutant *P23H-RFP/+* rods, the mislocalized P23H-hRho-RFP protein accumulated in the OPL in a similar pattern as in P30 mutant rods (Fig. 1G; Fig. S1H,I); although, interestingly, the morphology of the P90 accumulations appeared less densely packed and more dilated compared to P30 examples. There was no Rho staining in the OPL of WT P90 rods (Fig. S1J). Prominent Rho puncta were detected in 24.4±5.9% (mean±s.d., *N*=3 mice) of all P90 P23H-RFP/+ rod spherules imaged with SIM. In individual P90 mutant spherules, large P23H-hRho-RFP puncta were again localized inside the spherule cytoplasm, typically close to the ribbons, and were often observed in a swirling pattern around an empty gap in fluorescence. Thus, based on our SIM analysis, large cytoplasmic puncta of mutant P23H-hRho-RFP protein occur in ∼25% of the spherules in the *P23H-RFP/+* rods at P30, and this phenotype persisted in the surviving mutant rods at P90.

Our SIM results, combined with our previous findings, suggest that the ER is localized not only in mouse rod photoreceptor cell bodies but also in the presynaptic spherules. Whereas previous studies localized the ER throughout the IS, ONL and OPL in mouse retinas (Agrawal et al., 2017; Babai et al., 2010; Chen et al., 2015; Krizaj, 2005), we validated ER extension into the rod spherule cytoplasm using immunolabeling with Sec61β, an ER marker, and SIM. In both P30 WT and P23H-RFP/+ retinas, Sec61β ER labeling was distributed throughout the photoreceptor inner compartments (Fig. 1H), but in the *P23H-RFP/+* retinas, larger Sec61β^+^ puncta were localized in the IS and OPL, corresponding to the fluorescent ER accumulation in those mutant rods (Fig. 1I). In higher-magnification views of the OPL, ER immunofluorescence was clearly localized within the PSD95^+^ rod spherules in both genotypes, demonstrating that the ER network in rod photoreceptors extends from the IS to the rod presynaptic cytoplasm.

### P23H-Rho synaptic mislocalization does not cause ultrastructural defects in rod synaptic ribbons

**Fig. 2.**
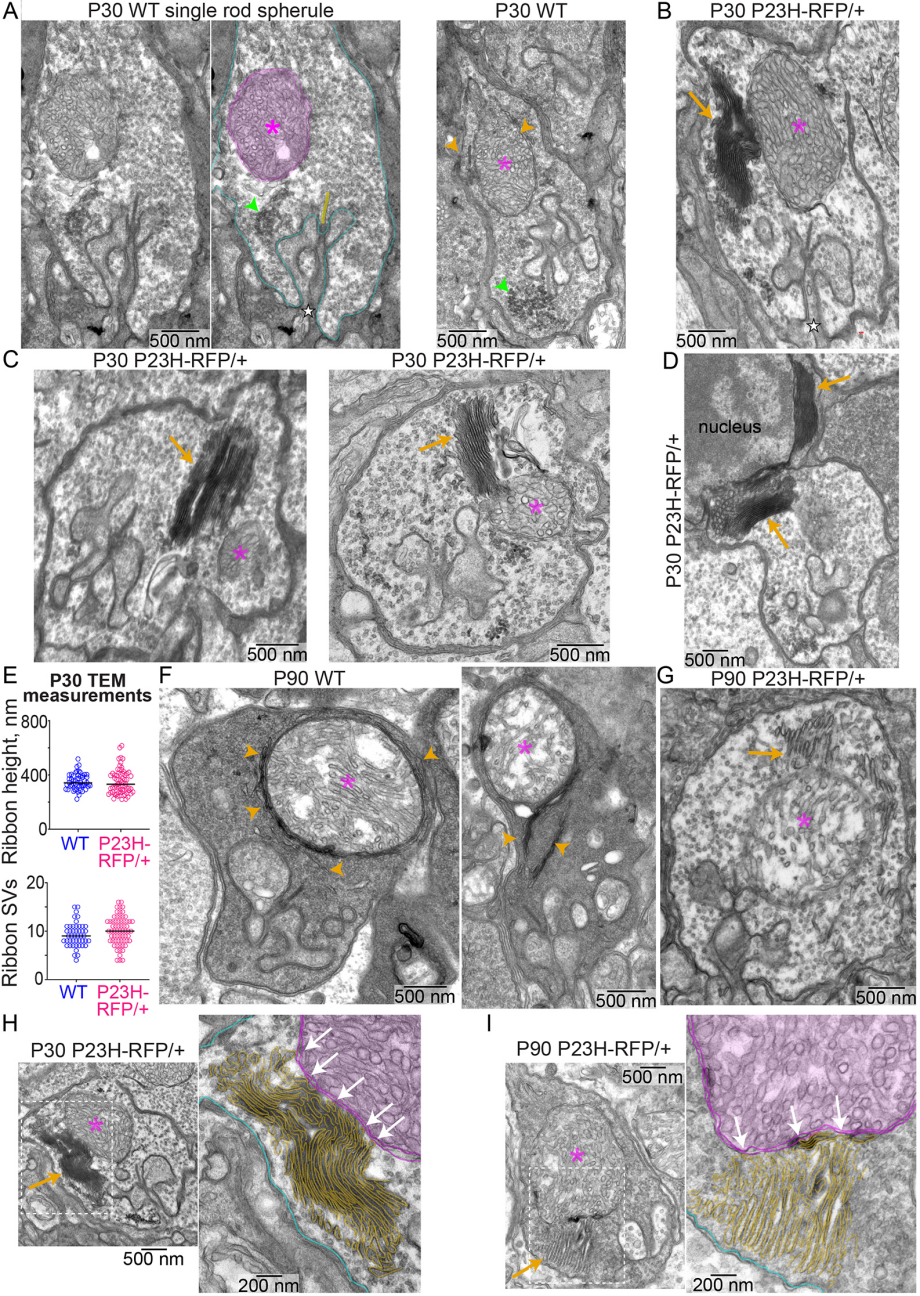
**P23H-Rho-RFP mislocalization does not cause ultrastructural defects in rod synaptic ribbons.** (A) Transmission electron microscopy (TEM) images of WT rod spherules at P30. Middle image is the annotated version of the left image (cyan, spherule plasma membrane; yellow, synaptic ribbon). In the right image, ER-like membranes are wrapped around the mitochondrion. (B) TEM image of a *P23H-RFP/+* rod spherule at P30. ER-like membrane stacks are localized near the mitochondrion and extend into the cytoplasm. (C) Additional TEM images of P30 *P23H-RFP/+* rod spherules with mitochondria-associated ER-like membrane stacks. (D) TEM image of a P30 *P23H-RFP/+* R2 rod spherule. (E) Top: ribbon height measurement graph from P30 TEM images. For WT, *n*=53 ribbons, *N*=3 mice; for *P23H-RFP/+*, *n*=68 ribbons, *N*=3 mice. Bottom: ribbon-associated synaptic vesicle (SV) count graph from P30 TEM images. For WT, *n*=46 ribbons, *N*=3 mice; for *P23H-RFP/+*, *n*=65 ribbons, *N*=3 mice. There are no significant differences (*P*≥0.05) based on an unpaired two-tailed *t*-test. (F) TEM images of P90 WT spherules. (G) TEM image of a P90 *P23H-RFP/+* spherule with expanded ER. (H,I) Magnified examples of ER-mitochondria contact sites (white arrows) in *P23H-RFP/+* spherules at P30 (H) and P90 (I). ER membranes are traced in gold, the mitochondrial membranes are traced in magenta, and the plasma membranes are traced in cyan. Throughout, magenta asterisks indicate mitochondria, green arrowheads indicate endocytosed vesicles, orange arrowheads indicate ER-like membranes, and orange arrows indicate ER-like membranes. Experiments were replicated in triplicate.

Based on the observation throughout our SIM analysis that mislocalized P23H-hRho-RFP occupied a large portion of the rod spherule cytoplasm, we hypothesized that this large amount of mislocalized protein could disrupt the rod presynaptic machinery on an ultrastructural level. In transmission electron microscopy (TEM) images of P30 WT mouse retinas, rod spherules have a distinct plasma membrane that surrounds the invaginating post-synaptic neurites, an electron-dense presynaptic ribbon that extends from the active zone, a cytoplasm filled with synaptic vesicles and a large mitochondrion (Fig. 2A). In many spherules, clusters of electron-dense endocytic vesicles denser than other cytoplasmic synaptic vesicles were observed (green arrowheads in Fig. 2A and Fig. S2A) (Fuchs et al., 2014). Additionally, more irregular membranes were found surrounding the spherule's mitochondrion (orange arrowheads in Fig. 2A and Fig. S2A), in a manner similar to ER membranes previously observed around mitochondria in other neurons (Wu et al., 2017) and in cat cone presynaptic pedicles (Lovas, 1971). Most P30 *P23H-RFP/+* mutant rod spherules had normal TEM ultrastructure, except in cases where electron-dense bundles of folded membranes were observed within the spherule cytoplasm (Fig. 2B,C, orange arrows). In some *P23H-RFP/+* rod spherules, the dense ER membrane stacks were less compact or potentially discontinuous (Fig. S2B); however, in most spherules, some portion of the ER membrane stacks closely localized near the spherule's mitochondrion. In one P30 P23H-RFP/+ R2 spherule, in which the spherule cytoplasm was continuous with the rod cell body, accumulated stacks of ER appeared to spill over from the cell body cytoplasm into the spherule cytoplasm (Fig. 2D).

The ultrastructure of the stacked membranes in *P23H-RFP/+* mutant rod spherules matches the semi-organized membrane stacks observed with TEM in the IS layer of *P23H-RFP/+* retinas (Robichaux et al., 2022), and they also match the localization and relative size of the P23H-Rho-RFP puncta observed in Fig. 1. Immuno-electron microscopy (EM) was used to localize Rho (with 1D4 immunolabeling) in the same stacked membranes of P30 *P23H-RFP/+* spherules and in the ER surrounding rod nuclei (Fig. S2C,D). Therefore, we conclude that these are expanded stacks of ER membranes within *P23H-RFP/+* spherules that are filled with mislocalized and accumulated P23H-hRho-RFP protein. Despite these large ER accumulations, there appeared to be no other major ultrastructural changes to the spherules or the synaptic ribbons in our P30 *P23H-RFP/+* TEM data. To confirm that the ribbon ultrastructure was unaffected, we measured the ribbon height and quantified the number of synaptic vesicles (SVs) associated with the ribbons in TEM images of P30 WT and *P23H-RFP/+* spherules, and no statistically significant differences were found (Fig. 2E).

The TEM ultrastructure of P90 WT rod spherules was similar to that of P30 WT rod spherules; however, there were more noticeable ER-like membranes surrounding the WT spherule mitochondria at this age (Fig. 2F). Based on our collective observations, we conclude that the irregular membranes surrounding the spherule mitochondria in P30 and P90 rod spherules are part of an endogenous network of ER within the spherule cytoplasm. ER-like membranes were also found in a WT rod axon (Fig. S2E) and spanning from the nucleus into a WT R2 spherule (Fig. S2F). P90 *P23H-RFP/+* mutant spherules also had expanded ER membranes that appeared tethered to the mitochondria (Fig. 2G). In higher-magnification TEM images of P30 and P90 *P23H-RFP/+* mutant spherules, multiple possible ER-mitochondrion contact sites were identified (Fig. 2H,I).

### Adeno-associated virus (AAV) overexpression of P23H-Rho and R135L-Rho in WT rods leads to synaptic mislocalization

To test whether the P23H-Rho mutation causes mislocalization in the OPL of rod photoreceptors with a non-disease, healthy genetic background, AAVs expressing P23H-hRho-TagRFP-T (P23H-hRho-RFP) or R135L-hRho-EGFP (R135L-hRho-GFP) mutant Rho fusions driven by the rod-specific minimal mouse opsin promoter (Pawlyk et al., 2005) were subretinally injected in adult WT mice. The R135L-Rho mutant as a GFP fusion was previously shown to be mislocalized to the OPL when electroporated into WT rat rods (Hsu et al., 2015). While P23H-Rho is a class 2 autosomal dominant RP mutation that misfolds and causes ER retention, R135L-Rho is a class 3 RP mutation that causes mutant protein accumulation in the endocytic pathway (Aguilà et al., 2014; Athanasiou et al., 2018; Chuang et al., 2004). As controls, WT-hRho-RFP and WT-hRho-GFP AAVs were generated to express WT-Rho fusion proteins in WT rods. All AAV constructs include a C-terminal 1D4 tag fused in-frame after RFP or GFP.

**Fig. 3.**
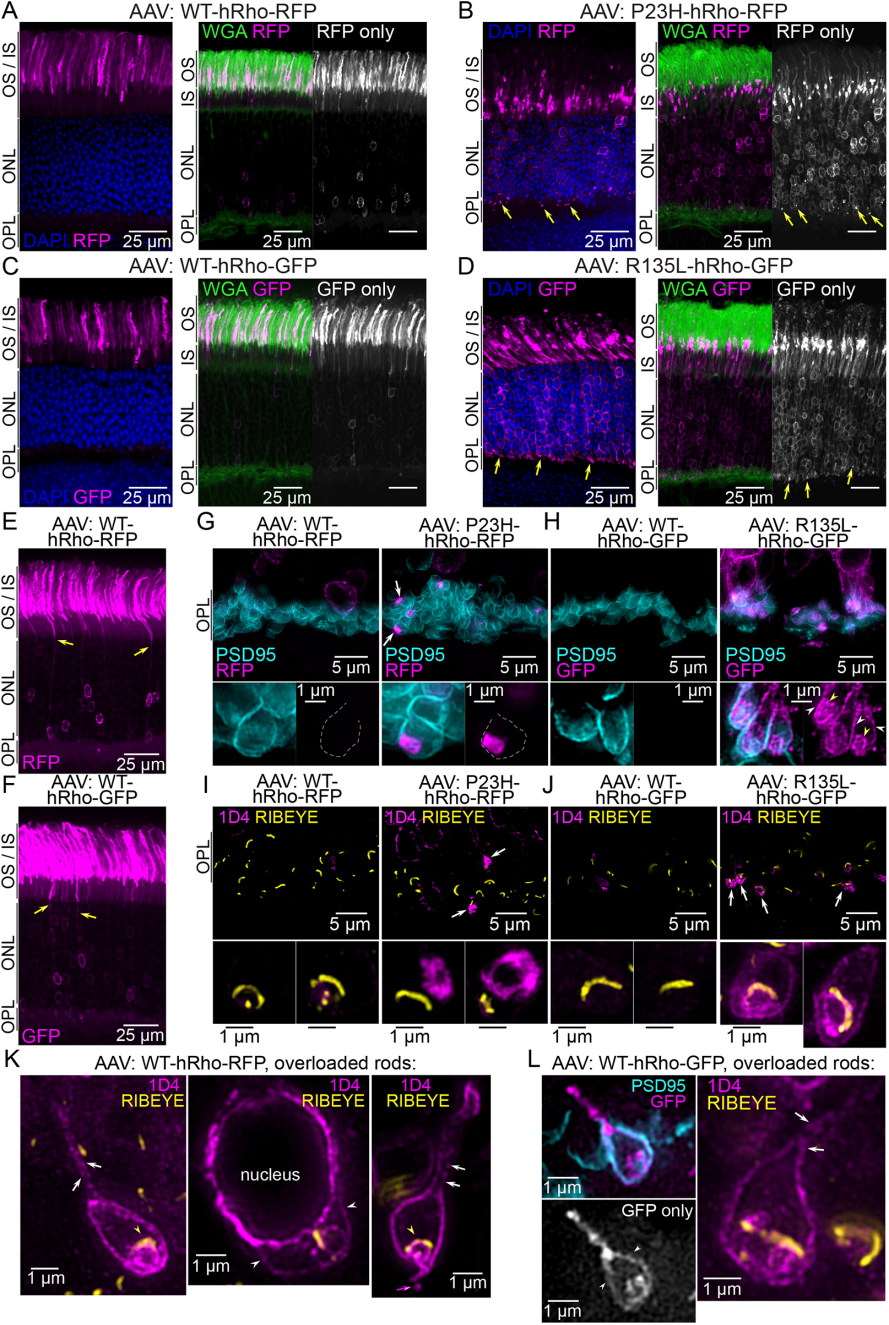
**Adeno-associated virus (AAV) overexpression of P23H-Rho and R135L-Rho in WT rods causes synaptic mislocalization.** (A-D) Confocal images of retinal sections from WT mice transduced with AAVs for rod-specific expression of the following Rho fusions: WT-hRho-RFP (A), P23H-hRho-RFP (B), WT-hRho-GFP (C) and R135L-hRho-GFP (D). Images on the left side show Rho fusion localization (magenta) in transduced rods with DAPI (blue) labeling. Right images show co-labeling with WGA (green). Yellow arrows indicate mutant Rho fusion OPL mislocalization. (E,F) AAV-infected retinal sections with rods overexpressing WT-hRho-RFP (E) or WT-hRho-EGFP (F) (magenta, yellow arrows). (G,H) SIM *z*-projection images of the OPL from 3-μm retinal cryosections from the same AAV conditions as in A-D. Rho fusion fluorescence is magenta, and PSD95 immunolabeling (cyan) was used to identify rod spherules. Single-spherule examples of each AAV-driven Rho fusion are shown below with PSD95 co-labeling (left) and the Rho-RFP/EGFP signal only (right). In G, white arrows indicate P23H-hRho-RFP cytoplasmic accumulated puncta. Dashed gray lines outline the PSD95^+^ plasma membrane of the magnified spherules. In H, R135L-hRho-GFP mislocalizes at the spherule plasma membrane (white arrowheads) and internally (yellow arrowheads). (I,J) SIM *z*-projection images from thin 1-μm sections of retinas from the same AAV conditions as in A-D. These sections were co-immunolabeled for 1D4 (magenta) and RIBEYE (yellow). White arrows indicate mutant Rho fusion accumulations. Single-spherule examples from each AAV condition are enlarged below. (K) SIM images of WT-hRho-RFP overloaded rod spherules. 1D4^+^ WT-hRho-RFP (magenta) was localized along the plasma membrane of the axon and spherule (white arrows) and colocalized with the RIBEYE synaptic ribbons (yellow arrowheads). In the far-right example, 1D4^+^ WT-hRho-RFP appears to bud off from the presynaptic spherule (magenta arrow). (L) SIM images of WT-hRho-GFP AAV overloaded rod spherules. WT-hRho-GFP colocalizes with the PSD95^+^ rod spherule plasma membrane (white arrowheads) and localizes at the rod axon plasma membrane (white arrows). All experiments were repeated in triplicate (*N*=3 mice per AAV).

Three weeks after subretinal AAV injection, retinas were screened with fluorescence microscopy to identify areas of high infection and no injection damage. Compared to WT-hRho-RFP, which predominantly localized correctly to the OS in transduced WT rods, P23H-hRho-RFP was mislocalized as bright puncta in the IS, encircling the nuclei throughout the ONL, and as distinct puncta in the OPL in transduced rods (Fig. 3A,B). This mislocalization pattern phenocopies the *P23H-RFP/+* mice and demonstrates that the P23H-hRho-RFP mislocalization is caused by the P23H-Rho mutation and not by non-autonomous effects in the disease-model transgenic mouse rods. WT-hRho-GFP also predominantly localized to the OS layer in transduced WT rods, while R135L-hRho-GFP accumulated as bright puncta at the IS/OS junction and mislocalized in a less bright, but more consistent, diffuse pattern throughout the IS, ONL and OPL (Fig. 3C,D). Additionally, whereas WT Rho fusions were strictly localized in the OS in most WT transduced rods, there were some sporadic transduced rods with clear overexpression of WT-Rho fusion protein that mislocalized to the other photoreceptor cell layers (Fig. 3E,F).

Next, AAV-transduced WT retinas were imaged with SIM to visualize the mislocalization of the mutant P23H-hRho and R135L-hRho fusion proteins in single rod spherules on a subcellular scale. First, cryosections of AAV-transduced retinas were used to preserve the Tag-RFP-T and EGFP fluorescence and were co-immunolabeled for PSD95 to label rod spherule plasma membranes. In these samples, the mislocalized P23H-hRho-RFP in the OPL were bright puncta, and no consistently strong signal above the background in the OPL was detected in control WT-hRho-RFP transduced retinas (Fig. 3G). In single spherules, the P23H-hRho-RFP puncta were localized within the cytoplasm, which phenocopies the subcellular localization of mislocalized P23H-hRho-RFP puncta in the OPL of *P23H-RFP/+* retinas. R135L-hRho-GFP mislocalization was also bright and apparent in the OPL of AAV-transduced WT retinas compared to WT-hRho-GFP, which was not detected in the OPL for most transduced areas (Fig. 3H). In single spherules, mislocalized R135L-hRho-GFP did not accumulate internally like P23H-Rho-RFP. R135L-hRho-GFP colocalized at the plasma membrane with PSD95 and internally in a pattern that suggests filling of the spherule plasma membrane surrounding the postsynaptic invaginations (Fig. 3H, yellow arrowheads).

For enhanced SIM resolution, we prepared thin plastic sections of AAV-transduced retinas. In this case, the Rho fusions were immunolabeled with the 1D4 antibody along with RIBEYE co-immunolabeling of the rod synaptic ribbons. In these sections, the mislocalized P23H-hRho-RFP proteins were visualized in a swirling pattern near the synaptic ribbon (Fig. 3I), again phenocopying the mislocalization pattern described in *P23H-RFP/+* retinas (Fig. 1F,G). R135L-hRho-GFP, on the other hand, was localized at the spherule plasma membrane and internally, partially colocalized with the synaptic ribbon (Fig. 3J), again suggesting that R135L-hRho-GFP fills in the invaginating plasma membrane. Among all WT retinas infected with either WT-hRho fusion, we observed a few examples of 1D4 staining in the OPL in sporadic cells with dramatically overexpressed levels of WT-Rho. In these overloaded rod spherules, WT-hRho-RFP and WT-hRho-GFP were mislocalized along the axons and throughout the spherule plasma membrane, including within the plasma membrane surrounding the synaptic invaginations (Fig. 3K,L) in a pattern similar to R135L-hRho-GFP mislocalization.

### P23H-Rho-RFP mislocalization causes specific changes in the abundance of some rod presynaptic proteins

**Fig. 4.**
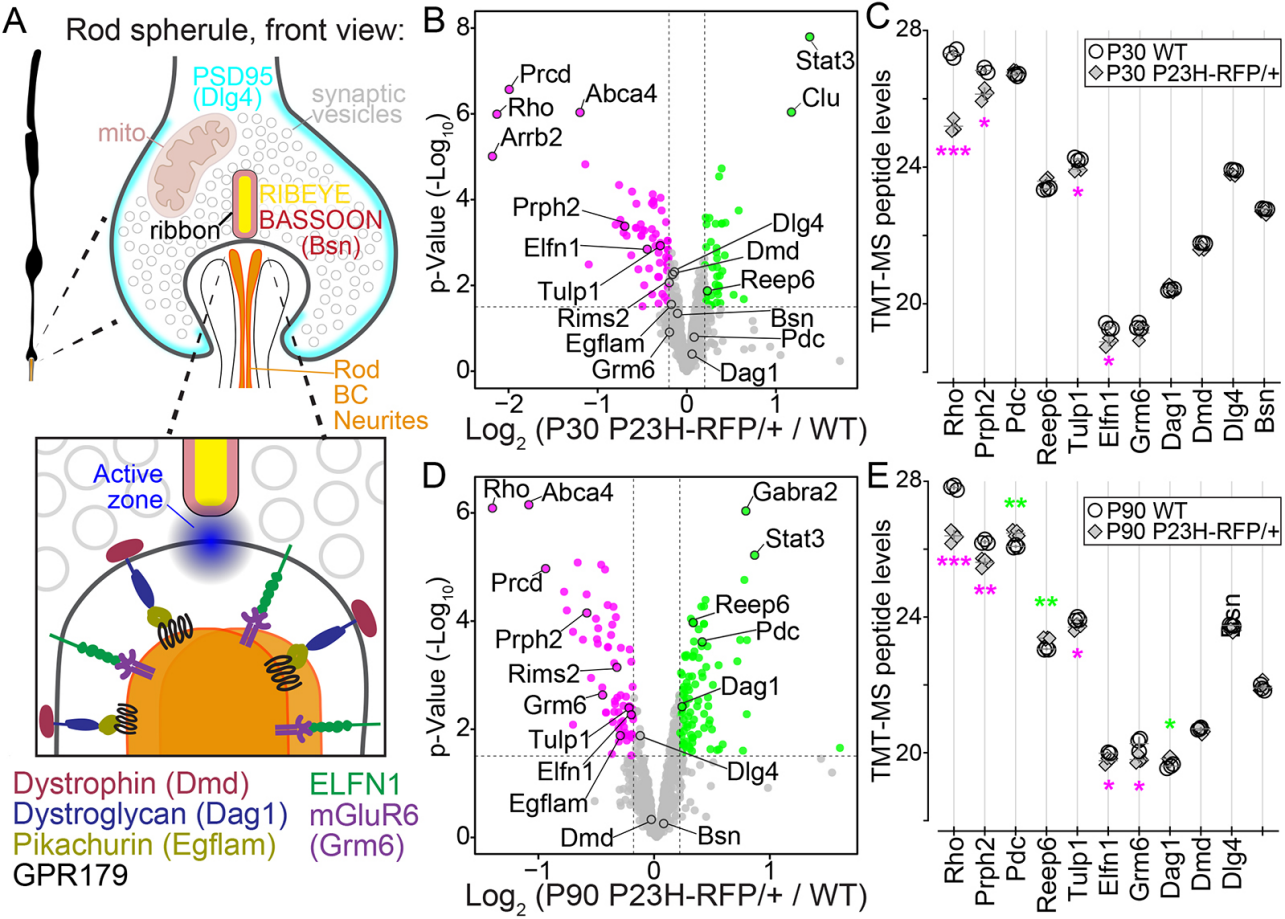
**Photoreceptor and synaptic protein abundance changes are found in *P23H-RFP/+* retinas with tandem mass tag-mass spectrometry (TMT-MS) proteomics.** (A) Diagram of the front view of a rod spherule (top) and a magnified view of the active zone and the synaptic cleft (bottom) to highlight the approximate localizations of rod synaptic proteins, including key trans-synaptic protein complexes. (B) Volcano plot of TMT-MS relative peptide abundances for select photoreceptor and synaptic proteins (see Materials and Methods) in P30 *P23H-RFP/+* versus WT retinas. *x*-axis, log_2_ fold change values with a significance threshold of 0.2; *y*-axis, *P*-values (−log10) with a significance threshold of 1.5. Green points represent protein targets above the thresholds and magenta points represent targets below the thresholds. Annotated protein names are based on FASTA gene names. (C) Peptide abundance graph of select log2 peptide values from the P30 TMT-MS data in Fig. S3B. WT (*N*=3, open circles) were superimposed with *P23H-RFP/+* values (*N*=4, gray diamonds). Magenta asterisks indicate significant downregulation; green asterisks indicate significant upregulation. (D) TMT-MS volcano plot comparing relative peptide abundances in P90 *P23H-RFP/+* (*N*=4) and WT (*N*=3) retinas for the same protein list and plot parameters as in B. (E) Linear scale graph of select peptide values from the P90 TMT-MS data in Fig. S3B with the same formatting as in C. *P<0.055, **P<0.01, ***P<0.001 (unpaired two-tailed *t*-test).

Because the persistent accumulation of mutant P23H-Rho protein within large ER folds in the cytoplasm of *P23H-RFP/+* rod spherules caused no ultrastructural defects to the synaptic ribbons, we considered whether the distension of the ER membranes throughout *P23H-RFP/+* rods disrupted normal rod synaptic protein levels. In rod photoreceptor spherules, the ribbon is composed of structural proteins and cell-adhesion proteins that maintain their trans-synaptic connections with rod ON-type bipolar cells. These include ELFN1, which complexes with postsynaptic mGluR6 (Grm6) (Cao et al., 2015), and Dmd, Dag1 and pikachurin (Egflam), which together complex with postsynaptic GPR179 (Furukawa et al., 2020; Omori et al., 2012; Orlandi et al., 2018; Sato et al., 2008) (Fig. 4A).

To assess protein level differences between *P23H-RFP/+* and WT rods, tandem mass tag-mass spectrometry (TMT-MS) was performed on whole-retina samples at P30 and P90. At both ages, there were statistically significant peptide abundance changes in *P23H-RFP/+* retinas compared to age-matched WT controls (Fig. 4B,D; Fig. S3A). As expected, Rho peptides were downregulated in *P23H-RFP/+* mice at both ages, along with peptides for the OS-specific protein peripherin 2 (Prph2) and the trafficking regulator Tubby-related protein 1 (Tulp1) (Hagstrom et al., 1999, 2001) (Fig. 4B-E). Peptide abundance for phosducin (Pdc), another OS protein, was increased in P90 *P23H-RFP/+* retinas, along with peptides for receptor expression-enhancing protein 6 (Reep6), an ER protein (Agrawal et al., 2017), whereas peptides for the rod synaptic proteins Elfn1, Rims2 and mGluR6 (Grm6) were decreased (Fig. 4E). Interestingly, peptide abundance for the rod trans-synaptic receptor Dag1 was increased to a statistically significant level at P90 in *P23H-RFP/+* rods; however, there was no such increase for its binding partner, Dmd, at either age. TMT-MS peptide abundance differences for rod synaptic proteins such as Dmd may not have been detected owing to their expression in other synapses of the inner retina, which were present in our whole-retina samples (Wersinger et al., 2011). Overall, this is a limitation for interpreting whole-retina TMT-MS changes, which could reflect cell loss changes in the *P23H-Rho-RFP* model. Dmd protein isoforms (Dp427, Dp260, Dp140 and Dp71) and Dag1 protein levels were also not changed in western blots from P30 *P23H-RFP/+* retinas compared to those from age-matched WT retinas, while only Dp71 and Dp140 were increased at P90 (Fig. S3B).

**Fig. 5.**
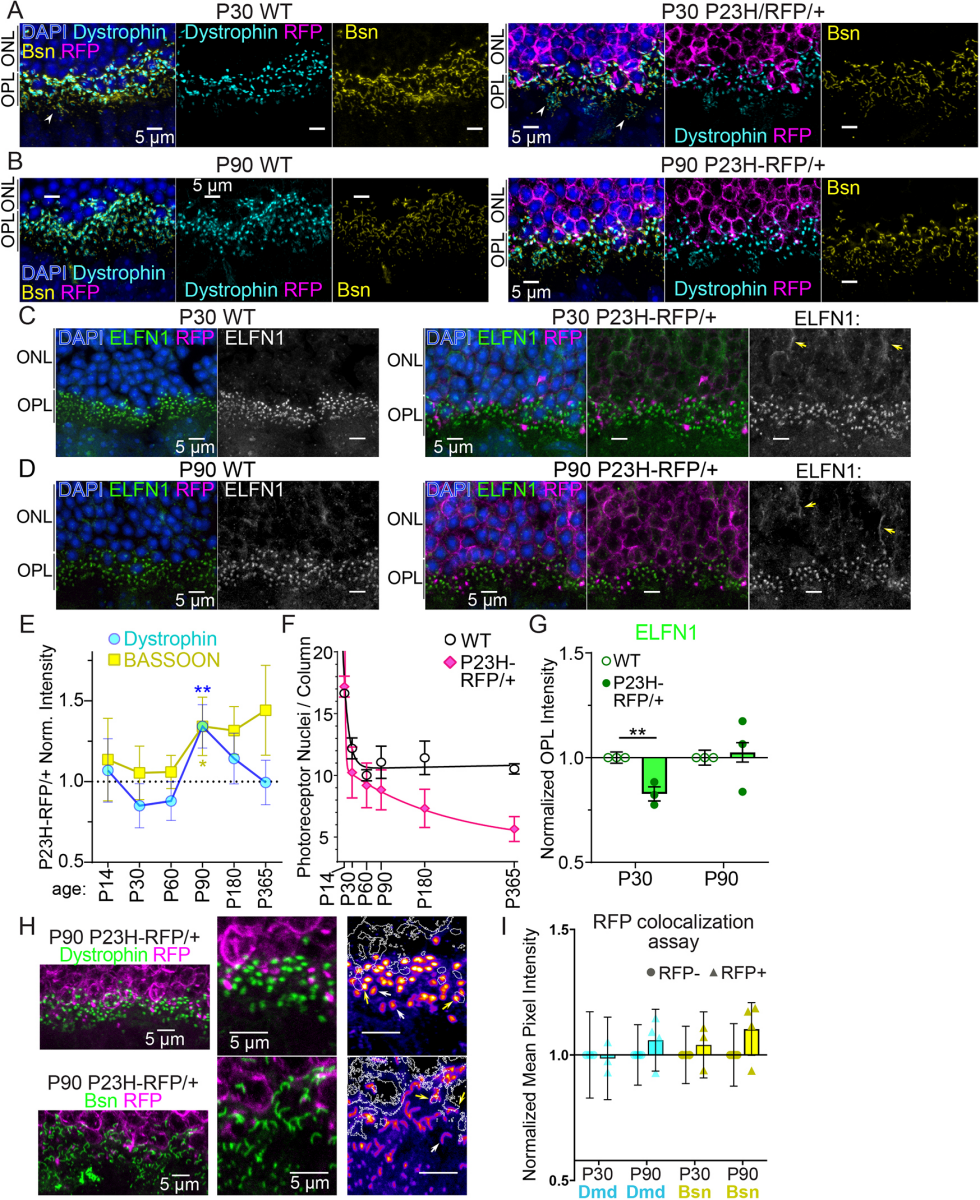
**Quantitative confocal imaging analysis of synaptic protein abundance changes in *P23H-RFP/+* mice.** (A,B) Example confocal *z*-projections from WT (*N*=3 mice) and *P23H-RFP/+* (*N*=3 P30 mice, *N*=4 P90 mice) retinal cryosections at P30 (A) and P90 (B) focused on regions of the lower/proximal ONL and OPL with dystrophin (Dmd; cyan) and Bsn (yellow) immunolabeling and DAPI counterstaining (blue). RFP fluorescence (magenta) was detectable only in the ONL and OPL of the *P23H-RFP/+* sections. White arrowheads indicate cone synapses. (C,D) Confocal *z*-projections for WT and *P23H-RFP/+* retinal cryosections at P30 (C) and P90 (D) with ELFN1 immunolabeling (green) and DAPI nuclear staining (blue). Yellow arrows indicate mislocalized strings of ELFN1. (E) Time course plot of Dmd (blue) and Bsn (yellow) normalized puncta intensity measurements from *P23H-RFP/+* retinas. Raw data image examples for timepoints P14 (*N*=3 mice, each group), P60 (*N*=3 WT mice, *N*=5 P23H-RFP/+ mice), P180 (*N*=3 mice, each group) and P365 (*N*=3 mice, each group) are provided in Fig. S4E. (F) Time course plot of DAPI^+^ photoreceptor nuclei per column counted from both WT and *P23H-RFP/+* in confocal images analyzed throughout the puncta analyses. Two-phase decay curve fits were added in GraphPad Prism. (G) Graph of normalized ELFN1 OPL intensities between WT and *P23H-RFP/+* retinas at P30 and P90 (*N*=3 mice, each group at each age). (H) Example confocal images from *P23H-RFP/+* retinas depicting RFP fluorescence (magenta) colocalized with either Dmd (top, green) or Bsn (bottom, green) immunolabeling. In magnified views, Dmd and Bsn intensities are heat map pseudocolored and superimposed with the RFP signal as white outlines. White arrows indicate RFP^+^ Dmd/Bsn colocalizations; yellow arrows indicate RFP^−^ Dmd/Bsn examples. (I) Graph of averaged puncta intensity values from the P90 *P23H-RFP/+* RFP colocalization assay for Dmd (cyan) and Bsn (yellow). Bars, mean values; error bars, s.d. Significance determined using unpaired two-tailed *t*-tests. Experiments were repeated in triplicate. **P<0.01.

To identify photoreceptor-specific protein changes, quantitative confocal microscopy was used to evaluate rod synaptic protein level changes specifically in the OPL of *P23H-RFP/+* and age-matched WT retinas. In confocal fluorescent images, immunolabeled Dmd and ELFN1 localized as bright puncta in the OPL among the mislocalized P23H-Rho-RFP, while Bsn immunolabeled the horseshoe-shaped synaptic ribbons (Fig. 5A-D). Dmd and Bsn were also localized in cone pedicle synapses (Fig. 5A, arrowheads). ELFN1 is specific to adult rod spherules (Cao et al., 2015, 2020), although we consistently observed an above-background ELFN1 immunofluorescence signal in the ONL of *P23H-RFP/+* retinas at P30 and P90 (Fig. 5C,D; Fig. S4A,B).

For Dmd and Bsn, a single-spherule puncta intensity analysis was performed using confocal imaging to evaluate a time course from P14 to P365, and P90 was the only timepoint at which there was a statistically significant, ∼30% increase for these proteins in *P23H-RFP/+* rods (Fig. 5E; Fig. S4C-E). There were, however, consistently higher Bsn levels at older timepoints. DAPI^+^ nuclei were counted in all P14-P365 replicate mice in this analysis and plotted in Fig. 5F, and the rate of photoreceptor degeneration in *P23H-RFP/+* mice, which plateaus at late stages, closely matches previous measurements (Robichaux et al., 2022). Notably, this is a difference from untagged P23H/+ knock-in mouse retinas, which eventually exhibit near-complete photoreceptor degeneration (Brooks et al., 2024). Interestingly, the P90 intensity increases for Dmd and Bsn correspond to the approximate timepoint at which photoreceptor loss in *P23H-RFP/+* retinas plateaus. ELFN1 is rod specific in the OPL; therefore, ELFN1 whole-layer OPL immunofluorescence intensities were compared between *P23H-RFP/+* retinas and age-matched WT mice because there was no interfering cone signal. At P30, ELFN1 levels in the OPL were significantly reduced in *P23H-RFP/+* retinas, but there was no reduction at P90 (Fig. 5G).

Because the RFP^+^ signal in the *P23H-RFP/+* OPL confocal images showed bright, easily detectable puncta, an additional analysis was performed comparing the intensities of Dmd and Bsn single-spherule signals that were either colocalized with RFP puncta (RFP^+^) or not (RFP^−^) in the OPL of P30 and P90 of *P23H-RFP/+* retinas (Fig. 5H). There were no statistically significant RFP^+^ versus RFP^−^ differences in the aggregated data (Fig. 5I). This result indicates that synaptic proteins are disrupted in all *P23H-RFP/+* rods, possibly due to P23H-Rho mislocalization in the ER throughout all the inner rod compartments, not just to ER accumulation in the spherules.

**Fig. 6.**
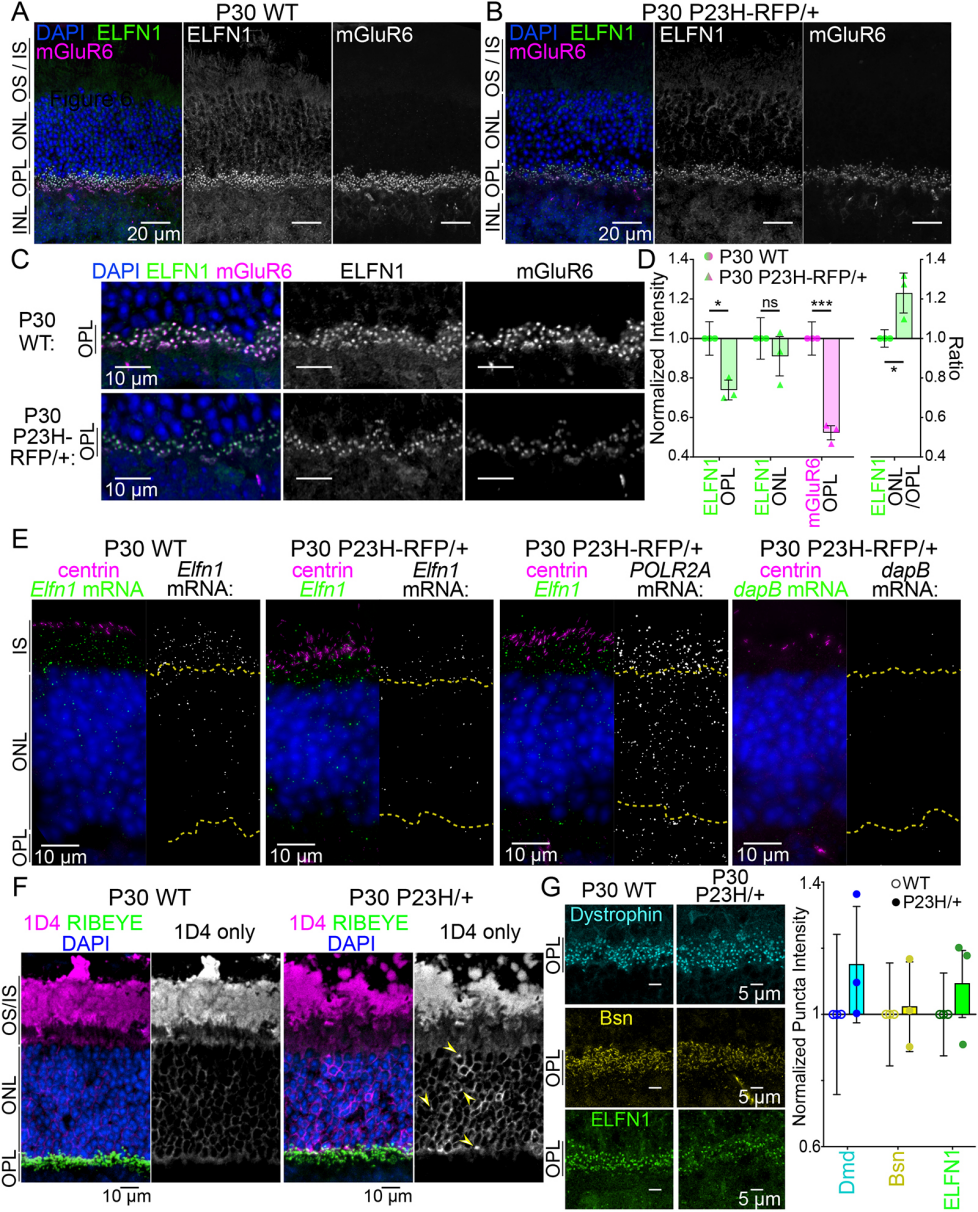
**ELFN1 protein distribution is altered in *P23H-RFP/+* retinas.** (A,B) Confocal *z*-projection images of P30 WT (A; *N*=3 mice) and P30 *P23H-RFP/+* (B; *N*=3 mice) retinal cryosections immunolabeled for ELFN1 (green) and mGluR6 (magenta) and counterstained for DAPI (blue). (C) Magnified confocal images with the same labeling focused on the OPL. Staining, acquisition and intensity settings were matched throughout A-C between the P30 WT and P30 *P23H-RFP/+* sections. (D) Normalized intensity graph (left) based on layer-specific intensity measurements for ELFN1 (green) and mGluR6 (magenta). Values were aggregated from replicate WT versus *P23H-RFP/+* experiments. On the right, ratios of ELFN1 intensity ONL/OPL intensities are plotted. (E) Example RNAScope SIM *z*-projections images for *Elfn1* mRNA (green) in P30 WT (*N*=3 mice) and P30 *P23H-RFP/+* (*N*=3 mice) retinas*,* alongside example SIM images probed for *POLR2A* (positive control) and *dapB* (negative control) mRNAs (P30 WT control images are in Fig. S5C). Sections were co-immunolabeled for centrin (magenta) to label connecting cilia and counterstained with DAPI (blue). Yellow dashed lines indicate the IS:ONL and ONL:OPL boundaries based on the DAPI staining. (F) Confocal images of P30 WT and *P23H/+* (right) retinas labelled for 1D4 (magenta), RIBEYE (green), and DAPI (blue) after antigen retrieval demasking. Yellow arrows indicate mislocalized Rho in the *P23H/+* ONL. (G) Graphed normalized puncta intensities for dystrophin (cyan), Bsn (yellow) and ELFN1 (green) from P30 WT and *P23H/+* OPLs. Bars, mean values; error bars, s.d. Significance determined using unpaired two-tailed *t*-tests. Experiments were repeated in triplicate. ns, not significant; *P<0.055, ***P<0.001.

We next evaluated the above-background ELFN1 immunofluorescence in the ONL that was observed in *P23H-RFP/+* retinas (Fig. 5C,D; Fig. S4A,B) by quantifying ELFN1 and mGluR6 localization throughout the outer retina. In P30 *P23H-RFP/+* and age-matched WT retinas, ELFN1 and mGluR6 immunofluorescence were predominantly localized as overlapping puncta in the OPL, and ELFN1 signal was detected in the ONL of both genotypes (Fig. 6A,B; Fig. S5A,B). Upon analysis of the OPL in these co-labeled sections, there was a decrease in ELFN1 and mGluR6 spherule puncta labeling in *P23H-RFP/+* OPLs (Fig. 6C) but no evidence of mGluR6 mislocalization, which was previously shown in *Elfn1* knockout mice (Cao et al., 2015). Layer-specific pixel intensity measurements from these confocal images confirmed reductions in ELFN1 (∼25% decrease) and mGluR6 (∼50% decrease) in the *P23H-RFP/+* OPL compared to that of WT controls (Fig. 6D; Fig. S5C). Interestingly, ELFN1 in the ONL was not different between *P23H-RFP/+* and WT retinas; however, there was a statistically significant, ∼20% increase in the ONL/OPL ratio of ELFN1 in *P23H-RFP/+* retinas (Fig. 6D), indicating a disruption in the distribution of ELFN1 in *P23H-RFP/+* mutant rods.

Given this protein distribution shift, *Elfn1* mRNA localization and abundance were evaluated using RNAScope fluorescence mRNA detection combined with SIM. With this method, *Elfn1* mRNAs were visualized as bright puncta localized throughout the IS and the ONL photoreceptor compartments in both P30 WT and P30 *P23H-RFP/+* retinas (Fig. 6E), demonstrating that *Elfn1* mRNA distribution was not grossly altered by the protein mislocalization and ER accumulation in *P23H-RFP/*+ rods at P30. Notably some *Elfn1* mRNA puncta were localized to the OPL, but these were sporadic and inconsistent. A positive control probe (*POLR2A*) targeting common housekeeping genes and a negative control probe (*dapB*) were analyzed for comparison (Fig. 6E; Fig. S5C). RNAScope puncta were counted to quantify any *Elfn1* mRNA abundance changes in the SIM data, and while *Elfn1* mRNA counts were higher in the IS layer and reduced in the OPL in *P23H-RFP/+* versus WT P30 retinas in aggregated data (Fig. S5D), the counts were variable in the data among the *P23H-RFP/+* replicates (Fig. S5E). *POLR2A* positive control mRNA counts were reduced in the distal ONL (dONL) and OPL in *P23H-RFP/+* retinas (Fig. S5F), indicating the possibility of a broader, cellular disruption to normal mRNA levels in *P23H-RFP/+* rods.

Next, rod synaptic protein level changes were analyzed in the non-fusion *P23H-Rho/+* knock-in mouse model (Sakami et al., 2011) as a comparison to the *P23H-RFP/+* model. In *P23H/+* mouse retinas, Rho mislocalized around photoreceptor cell bodies of the ONL, but this was only detectable with antigen retrieval (Vasudevan et al., 2024). Here, we applied antigen retrieval unmasking and validated that 1D4 Rho immunolabeling was mislocalized most evidently in the photoreceptor cell bodies in P30 *P23H-Rho/+* retinas (Fig. 6F). Then, single-spherule puncta intensity analysis was performed to evaluate Dmd, Bsn and ELFN1 in the *P23H/+* OPL; however, we found no differences (Fig. 6G), indicating that the synaptic protein changes in *P23H-RFP/+* rods may be due to the unique mislocalization and intracellular accumulation that spans the IS, ONL and OPL compartments in those rods.

### In rd10 rods, Rho is mislocalized along the spherule plasma membrane and presynaptic ELFN1 levels are disrupted

As another comparison with our *P23H-RFP/+* model, we analyzed rd10 mice, an RP model known to exhibit synaptic mislocalization of non-mutated WT Rho (Barhoum et al., 2008; Zhao et al., 2015). Rd10 rod degeneration begins around P18 and peaks at ∼P20 (Gargini et al., 2007; Jae et al., 2013; Wang et al., 2015; Zhao et al., 2015). Despite this early onset of degeneration, rd10 mutant rod ribbon synapses were shown to be morphologically normal (Grossman et al., 2009). Here, confocal imaging of Rho immunofluorescence was used to examine rd10 retinas, which appeared relatively normal at P16, a pre-degeneration stage (Fig. S6A), but displayed degenerative morphology by P20 (Fig. S6B). Based on our imaging, rd10 retinas at P16 and P20 had Rho mislocalization to the IS, ONL and OPL (Fig. S6A,B).

SIM was used to examine the subcellular localization of Rho mislocalization in rd10 mice at P16 and P20. Retinas were immunolabeled with the 4D2 monoclonal Rho antibody (Hicks and Molday, 1986), a PSD95 fluorescent nanobody and a RIBEYE antibody. Although Rho immunofluorescence was detected in the IS and ONL of both P16 WT and rd10 retinas, the amount of Rho throughout the IS and ONL appeared higher in rd10 rods than in WT rods. Rho was also clearly present in the rd10 OPL at P16 (Fig. S6D), which indicates Rho mislocalization at this age. In the IS of P16 WT rods, we detected clear IS plasma membrane labeling (Fig. S6F), while in P16 rd10 rods, mislocalized Rho was enriched in the Golgi-rich IS myoid region and along the IS plasma membrane (Fig. S6G). In the ONL of both P16 WT and rd10 rods, Rho immunofluorescence surrounded the rod nuclei and also localized to the edges of ∼0.5-μm-diameter projections corresponding to either the rod axons or the outer fibers that connect mouse rod ISs and cell bodies (Fig. S6H). In P20 rd10 retinas, Rho was also highly mislocalized in the IS compared to that in age-matched WTs; however, OPL mislocalization was not evident at P20 (Fig. S6E), possibly due to advanced degeneration or labeling penetration issues.

**Fig. 7.**
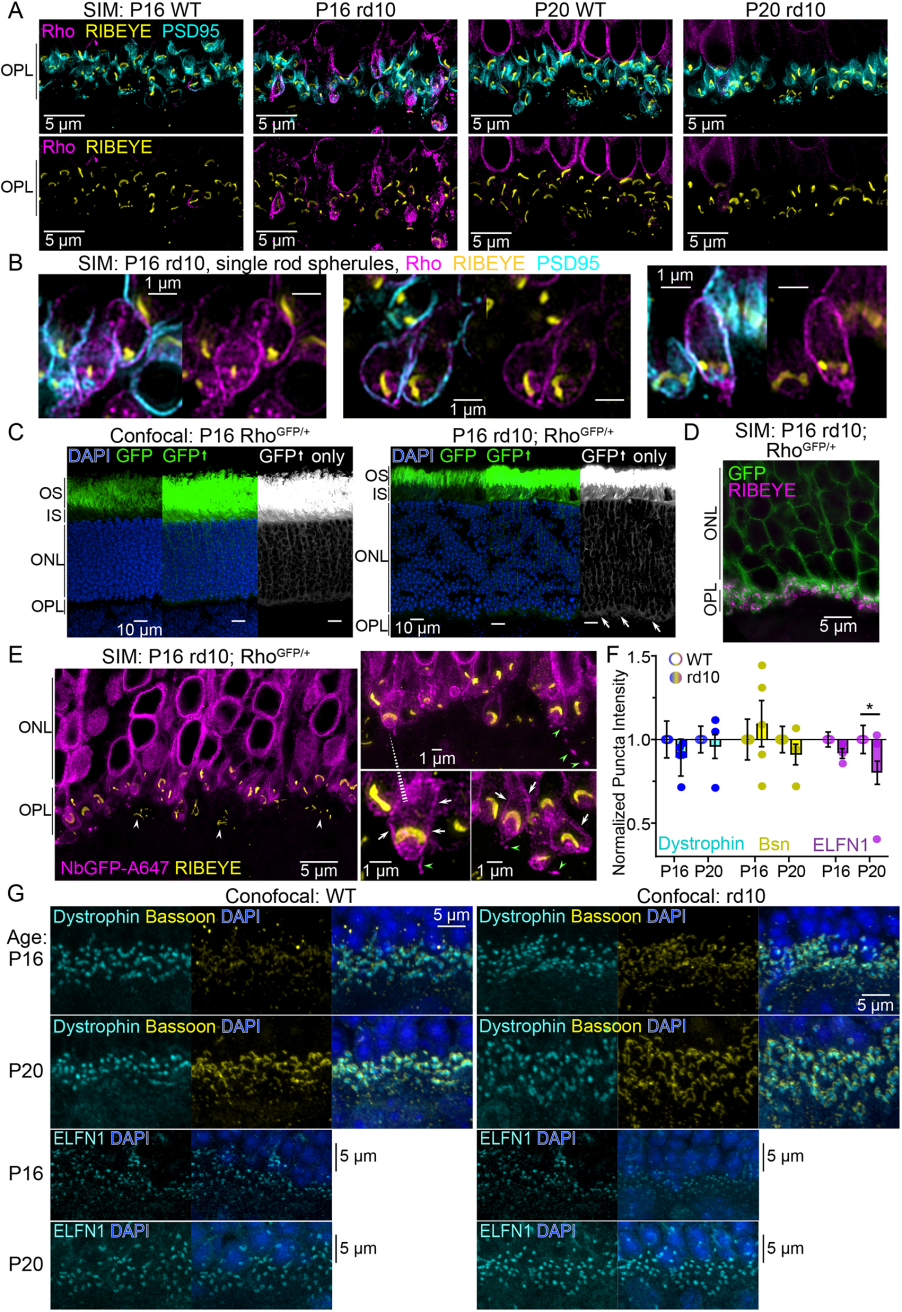
**Analysis of Rho mislocalization and presynaptic protein levels in rd10 RP mutant retinas.** (A) SIM images of the OPL in WT and rd10 mice at P16 and P20. Retinas were immunolabeled for 4D2 (Rho, magenta), RIBEYE (yellow) and PSD95 (cyan). In the WT OPL, the 4D2 signal is not consistently above background levels. (B) Magnified single-rod spherule SIM images from P16 rd10 retinas with the same labeling as in A. (C) Confocal *z*-projections of cryosections from Rho^GFP/+^ (left) and rd10; Rho^GFP/+^ (right) retinas depicting Rho-GFP (GFP, green) localization. Sections were counterstained with DAPI (blue). Grayscale images are GFP only with increased brightness to demonstrate Rho-GFP OPL mislocalization in rd10; Rho^GFP/+^ retinas (white arrows). (D) SIM image of a P16 rd10; Rho^GFP/+^ retinal cryosection immunolabeled for RIBEYE (magenta) to demonstrate mislocalized Rho-GFP overlap with RIBEYE^+^ ribbons. (E) SIM images of thin resin sections of P16 rd10; Rho^GFP/+^ retinas co-immunolabeled with a GFP nanobody (NbGFP-A647, magenta) and for RIBEYE (yellow). NbGFP-A647 labeling was specific for Rho-GFP because cone ribbons are present (white arrowheads) had no NbGFP-A647 staining. In magnified views of rd10; Rho^GFP/+^ spherules, Rho-GFP is localized in the OPL along the spherule plasma membranes (white arrows). Small Rho-GFP puncta were observed in the OPL extracellular space as if detached from the spherule membrane (green arrowheads). (F) Graph of aggregated normalized puncta intensities for dystrophin (cyan), Bsn (yellow) and ELFN1 (purple) from P16 and P20 WT (open circles) and rd10 (filled circles) retinas. Raw image examples are in Fig. S6G. Experiments were repeated in triplicate. Significance determined using unpaired two-tailed *t*-test (*P<0.05,) (G) Raw confocal image examples of WT and rd10 retinas at P16 and P20 used in F.

In SIM images focused on the OPL in age-matched WT and rd10 mice, Rho immunofluorescence signal was most abundant in P16 rd10 retinas, in which mislocalized Rho labeled many teardrop-shaped spherules surrounding the RIBEYE^+^ rod synaptic ribbons, whereas in P20 rd10 OPL, mislocalized Rho immunofluorescence was again less evident (Fig. 7A). Notably, in control P20 WT retinas, we only detected Rho in the bottom rows of nuclei in the ONL (Fig. 7A; Fig. S6E) likely due to limited antibody staining penetration into the IS and ONL in P20 WT rods that have more developed OSs, a phenomenon we previously described for staining in adult mouse rods (Haggerty et al., 2024). In high-magnification images of P16 rd10 rod spherules, mislocalized Rho was predominantly located along the spherule plasma membrane, colocalized with PSD95 (Fig. 7B; Fig. S6I). In some cases, the mislocalized Rho was also clearly localized along the axon that was continuous with the spherule plasma membrane; in other cases, Rho appeared to be mislocalized internally, within the spherule cytoplasm and partially colocalized with the RIBEYE^+^ ribbon (Fig. S6I). However, it was not possible to distinguish whether this signal represents Rho within the invaginating spherule plasma membrane, as seen for R135L-hRho-EGFP AAV-transduced rods (Fig. 3H,J) and adult rods overexpressing WT-hRho-RFP (Fig. 3K), or some other internal localization. Importantly, at this magnification, there was no detectable Rho immunofluorescence in P16 WT spherules (Fig. S6J).

To confirm this mislocalization pattern, rd10 mice were crossed with the *Rho-GFP-1D4* mice to generate a mouse homozygous for the rd10 mutation and heterozygous for Rho-GFP-1D4 knock-in fusion (rd10; Rho^GFP/+^). Using confocal imaging, Rho-GFP was mislocalized in the OPL in P16 rd10; Rho^GFP/+^ retinas compared to control Rho^GFP/+^ retinas (Fig. 7C); however, as with Rho immunofluorescence staining in P16 rd10 retinas (Fig. S6A,B), the mislocalized Rho-GFP was clearly not accumulated in the OPL. With SIM imaging of P16 rd10; Rho^GFP/+^ retinas, Rho-GFP fluorescence was first colocalized with the RIBEYE^+^ ribbons in the OPL (Fig. 7D), and then GFP nanobody staining was used to visualize Rho-GFP localization in single rod spherules. The Rho-GFP mislocalization pattern was again predominantly localized along the spherule plasma membrane, encasing the RIBEYE^+^ synaptic ribbons, with some potentially internal Rho-GFP that could be continuous with the Rho-GFP that was heavily localized in the ONL surrounding the rod nuclei (Fig. 7E). Additionally, we observed some Rho-GFP signal that branches away from the spherule into the extracellular matrix of the OPL (Fig. 7E, green arrowheads).

Finally, rd10 mice at P16 and P20 and age-matched WT controls were used to test for any Dmd, Bsn and ELFN1 protein level changes in the OPL caused by Rho mislocalization. Upon quantitative confocal analysis, the only statistically significant difference was a ∼20% reduction in rd10 ELFN1 mean puncta intensities at P20 (Fig. 7F,G). Therefore, synaptic proteins were generally less affected by Rho mislocalization in rd10 rods compared to *P23H-RFP/+* rods.

## DISCUSSION

**Fig. 8.**
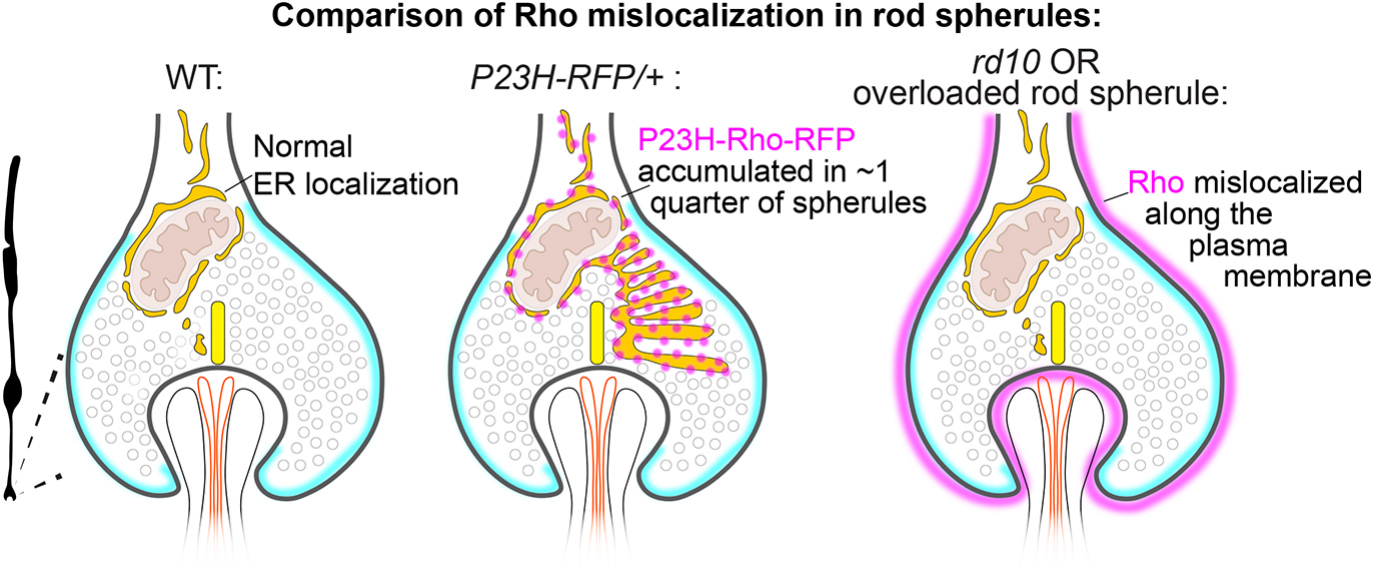
**Diagram comparing Rho mislocalization pattern differences in mutant rod spherules.** The spherule plasma membrane is highlighted in cyan. In WT spherules, ER (gold) wraps around the mitochondrion (tan) in the cytoplasmic space above the synaptic ribbon (yellow) and the synaptic cleft containing the postsynaptic neurites (ON-type bipolar cells, orange; horizontal cells, black). In approximately one-quarter of P23H-RFP/+ rod spherules, mutant Rho (magenta dots) accumulates in expanded ER (gold) in the cytoplasmic space . In rd10 rod spherules or WT spherules with overexpressed WT-Rho fusion protein or mutant R135L-hRho-GFP protein, Rho (magenta) mislocalizes along the spherule plasma membrane (cyan) such that the ER (gold) is putatively unaffected.

In this study, we found that P23H-Rho-RFP protein accumulated throughout rod cytoplasm, including in the presynaptic spherules of approximately one-quarter of rod photoreceptor neurons from *P23H-RFP/+* RP mutant mice. The intracellular accumulation of P23H-Rho-RFP did not disrupt synaptic ribbon ultrastructure or active zone synaptic vesicle density; instead, we found that some rod presynaptic protein levels were disrupted in these mutants. We also found three separate cases in which Rho mislocalized along the spherule plasma membrane: (1) in WT rods expressing RP mutant R135L-hRho-GFP (Fig. 3H,J), (2) in WT rods overloaded with WT-Rho-GFP/RFP fusion proteins (Fig. 3K,L), and (3) in rd10 RP mutant rods in which a PDE6β mutation disrupts proper Rho trafficking, which then overloads the rod plasma membrane (Fig. 7B,E). Thus, our findings provide new subcellular localization details of how different Rho mislocalization patterns differentially impact mouse rod presynaptic terminals (Fig. 8).

In the *P23H-RFP/+* mouse line used in this study, we show that ER accumulation of P23H-Rho-RFP extends throughout the rod inner compartments and into the OPL of ∼25% of rods. However, importantly, the large amount of accumulated P23H-Rho-RFP near these presynaptic ribbons found at both P30 and P90 does not drive *P23H-RFP/+* rod degeneration because the rate of degeneration does not correspond to the number of cells with this phenotype. We also used a newly generated *WT-RFP/+* mouse to demonstrate that *P23H-RFP/+* mislocalization to synaptic accumulations is specifically triggered by the P23H-Rho mutation (Fig. 1B,C), and we analyzed P30 *P23H/+* knock-in mouse retinas and found no *P23H/+* presynaptic protein changes like we did in *P23H-RFP/+* retinas. Previously, there were no detected changes to mGluR6 levels in 1-month-old *P23H/+* retinal sections (Leinonen et al., 2020). Based on these results, we conclude that the *P23H-Rho-RFP* mouse line has unique phenotypes, including synaptic protein differences, owing to ER retention of misfolded Rho protein, which is a known component of RP pathology (Chiang et al., 2015). Thus, *P23H-Rho-RFP* could be a valuable tool for investigating ER-related homeostatic adaptations and rod response mechanisms in future studies.

Throughout this study, we examined the differences in Rho mislocalization between the *P23H-RFP/+* retinas and other models with SIM, which enabled us to clarify the extent of the ER network in mouse rods. However, we note that the impact of cell degeneration on rod photoreceptor ER in P23H-Rho-RFP/+ mice, which could be an important factor, was not directly addressed in this study. An inter-compartmental rod ER network that reaches the presynaptic spherules was previously described in amphibian rods (Mercurio and Holtzman, 1982) and salamander rods (Chen et al., 2014, 2015). Mouse rods are morphologically different: the spherules are more segregated from the inner segment, such that mouse spherules each have a mitochondrion. A study in mice used sarco/endoplasmic reticulum Ca^2+^-ATPase (SERCA) immunostaining to establish that presynaptic ER Ca^2+^ release helps sustain prolonged depolarizing conditions in rods (Babai et al., 2010). Our TEM imaging confirms these observations, as we visualized distinct ER-mitochondria associations between the ER and the mitochondrial outer membrane in *P23H-RFP/+* spherules (Fig. 2H,I). Other TEM studies noted ER membranes wrapped around the mitochondria in WT mouse rod spherules (Johnson et al., 2007; Ladman, 1958). Based on this association, the mouse spherule ER may form functional mitochondria-ER contacts that could participate in regulating proper glutamate release, lipid exchange or Ca^2+^ signaling (Križaj, 2012; Tsuboi and Hirabayashi, 2021).

Among the synaptic protein abundance changes we discovered in *P23H-RFP/+* retinas, the depletion of ELFN1 in the OPL of P30 *P23H-RFP/+* rods was the most prominent finding (Fig. 5G and Fig. 6A-D). In our TMT-MS data, *P23H-RFP/+* ELFN1 peptide abundances were also reduced at P30 and P90 (Fig. 4C,E). Because ELFN1 localized as strings of fluorescence in the ONL from P30 *P23H-RFP/+* retinas throughout the images in Figs 5 and 6, resulting in a shift in ONL/OPL distribution of ELFN1, we hypothesize that ELFN1 protein trafficking is specifically disrupted by P23H-hRho-RFP accumulation. Expectedly, this ELFN1 deficit coincided with diminished postsynaptic mGluR6 puncta staining in the *P23H-RFP/+* OPL at P30 (Fig. 6C,D), because ELFN1 and mGluR6 form a critical trans-synaptic complex between rod spherules and ON-type bipolar cell dendritic tips.

Interestingly, at P90, *P23H-RFP/+* ELFN1 confocal immunofluorescence levels returned to WT levels (Fig. 5G), while Dmd and BASSOON levels were unchanged in P30, but then higher in P90, *P23H-RFP/+* retinas compared to WT control retinas (Fig. 5E; Fig. S4C,D). Because Dmd and BASSON are soluble proteins, we speculate that the overloaded *P23H-RFP/+* ER clogs the narrow rod compartments such that it disrupts the normal turnover of these proteins. Furthermore, in spherules, Dmd complexes with Dag1/Egflam (Sato et al., 2008), while Bsn forms essential protein complexes at the ribbon (Dick et al., 2003), and so it is possible that the formation of these complexes is disrupted by ER accumulation. P90 *P23H-RFP/+* rods were described as having adaptive mechanisms that enabled some degree of normal Rho trafficking to the OS and cell survival (Robichaux et al., 2022). Upregulation of presynaptic proteins may be caused by inefficient protein turnover or be the result of a mechanism used by surviving P90 *P23H-RFP/+* rods to preserve essential rod synapses during degeneration. In support of this, a previous study using *P23H/+* heterozygous knock-in mice demonstrated synaptic transcriptome increases at 3 months (∼P90), which was attributed to homeostatic responses in rod bipolar cells to increase their sensitivity to rod input (Leinonen et al., 2020).

In addition to the intracellular accumulation of P23H-hRho-RFP mutant protein, our visualization of mislocalized WT-Rho protein outlining the rod spherule plasma membrane either in WT rods overloaded with WT-Rho fusion protein (Fig. 3K,L) or in P16 rd10 rods (Fig. 7B,E) is informative about endogenous Rho trafficking dynamics. In our previous study, we used multiple labeling approaches with quantitative single-molecule imaging to show that Rho is located throughout the IS plasma membrane in WT/non-diseased mouse rods (Haggerty et al., 2024). Thus, Rho that is overloaded in the IS may disperse throughout the entire rod plasma membrane and into the rod spherule plasma membrane, including within the postsynaptic neurite invaginations. The mislocalized Rho in these overloaded regions may then be removed through endocytosis (Ropelewski and Imanishi, 2019) or exocytosis (Lewis et al., 2022), the latter of which we potentially observed here in the OPL in P16 rd10; RhoGFP/+ rods (Fig. 3K and Fig. 7E).

In summary, our study provides an in-depth examination of P23H-Rho-RFP accumulation in presynaptic rod spherules and evidence that some rod synaptic proteins are sensitive to these ER disruptions. An essential next step will be elucidating the trafficking mechanisms used for proteins like ELFN1 and Dag1, which both require precise post-translational glycosylations to properly form trans-synaptic interactions (Park et al., 2020; Sato et al., 2008). We previously found no evidence of Golgi in the OPL (Robichaux et al., 2022); however, it is unclear how synaptic proteins would be transported to the OPL from the IS Golgi or whether there is an alternative pathway that utilizes the rod's ER secretory network. Future studies that elaborate the secretory system for rod synaptic proteins will provide much-needed insight into subcellular disease mechanisms in rod neurons.

## MATERIALS AND METHODS

### Animals

The *P23H-hRho-TagRFPt* (*P23H-RFP*) and *Rho-GFP-1D4* mice (*Mus musculus*) were previously described (Haggerty et al., 2024; Robichaux et al., 2022) and were C57BL/6J. WT mice controls were C57BL/6J. The rd10 mice (The Jackson Laboratory) were also C57BL/6J and were crossed with *Rho-GFP-1D4* homozygotes to generate rd10;*Rho^GFP^* mice. The *P23H-Rho* knock-in mice (Sakami et al., 2011) used in this study were obtained backcrossed on a 129E background, and so the WT control mice used in Fig. 6F,G were also 129E. *WT-hRho-TagRFP-T* (*WT-RFP*) mice were generated using CRISPR gene editing in the Genetically Engineered Murine Model Core Facility, which is supported by the University of Virginia School of Medicine, Research Resource Identifier (RRID):SCR_025473. The sgRNA (AGTACTGTGGGTACTCGAAGTGG) and HDR donor (GCCACAGCCATGAATGGCACAGAAGGCCCTAACTTCTACGTGCCCTTCTCCAATGCGACGGGTGTGGTACGCAGTCCCTTCGAGTACCCACAGTACTACCTGGCTGAGCCATGGCAGTTCTCCATGCTGGCCGCCTACATGTTTCTGCTGAT) oligonucleotides were developed for correcting the H23 mutant codon (CAC) to the WT P23 (CCC; underlined) codon in *P23H-RFP* mice. CRISPR reagents were injected into fertilized zygotes from *P23H-RFP* homozygous male and female mice for germline genome integration. Founder mice were then crossed to C57Bl/6J WT mice. F1 progeny were screened to confirm that the P23H mutation was corrected by sequencing genomic DNA. There was an unexpected silent change in the genome for residue S22 (AGC to TCA), but this did not change the coded amino acid sequence of hRho. We also validated the *WT-RFP* knock-in with western blotting and fluorescence microscopy (Fig. 1B,C; Fig. S1A,B), and because validation was completed in the middle of this study, we continued using WT mice as controls for comparison to *P23H-RFP* mice throughout these experiments.

All mice were housed in 12 h light/dark conditions. Both sexes were used for experiments in the study unless otherwise noted. Mouse ages for imaging are denoted in the data. All experimental procedures using mice were approved by the Institutional Animal Care and Use Committee of West Virginia University (WVU) (approval #2102040326).

### Antibodies and labeling reagents

The following primary antibodies were used in this study: anti-1D4 (Millipore Sigma, MAB5356, RRID:AB_2178961); anti-4D2 (Millipore Sigma, MABN15, RRID:AB_10807045); anti-Dmd (immunofluorescence) (Abcam, ab15277, RRID:AB_301813); anti-Dmd (western blotting) (Proteintech, 12715, RRID:AB_10640422); anti-Dag1 (Proteintech, 66735, RRID:AB_2934490); anti-RIBEYE (Synaptic Systems, 192103, RRID:AB_2086775); anti-Bsn (Enzo, SAP7F407, RRID:AB_2313990); anti-TUBB5 (Millipore Sigma, MAB380, RRID:AB_2210541); anti-Sec61β (Cell Signaling Technology, D5Q1W, RRID:AB_2798555); anti-centrin, 20H5 (Millipore Sigma, 04-1624, RRID:AB_10563501); anti-ATP1B2 (Proteintech, 22338, RRID:AB_2879077); FluoTag-X2 anti-PSD95-Alexa647 nanobody (NanoTag Biotechnologies, N3702-AF647, RRID:AB_2936216). The anti-ELFN1 polyclonal antibody was provided by Dr Kirill Martemyanov (University of Florida Scripps Institute). The anti-mGluR6 polyclonal antibody was provided by Dr Melina Agosto (Dalhousie University) (Agosto et al., 2021).

The following secondary antibodies were used in this study: F(ab′)2-goat anti-rabbit Alexa 488 IgG (Invitrogen, A11070, RRID:AB_2534114); F(ab′)2-goat anti-mouse Alexa 488 IgG (Invitrogen, A11017, RRID:AB_2534084); F(ab′)2-goat anti-rabbit Alexa 647 IgG (Invitrogen, A21246, RRID:AB_2535814); F(ab′)2-goat anti-mouse Alexa 647 IgG (Invitrogen, A21237, RRID:AB_2535806); F(ab′)2-goat anti-mouse Alexa 555 IgG (Invitrogen, A21425, RRID:AB_2535846); IRDye800CW goat anti-rabbit IgG (LI-COR, 925-32211, RRID:AB_2651127); IRDye800CW goat anti-mouse IgG (LI-COR, 925-32210, RRID:AB_2687825). Wheat germ agglutinin (WGA) staining was performed with WGA-CF640R (Biotium, 29206) and WGA-Alexa-CF488A (Biotium, 29022).

### Retinal immunofluorescence

For immunofluorescence staining of mouse retinal cryosections for confocal microscopy, mouse eyes were enucleated, and the cornea, lens, and optic nerve were removed in ice-cold 4% (w/v) paraformaldehyde (PFA) fixative. Eyecups were fixed for an additional 15 min at room temperature and transferred to a 30% sucrose in 1× phosphate-buffered saline (1×PBS) solution for 2 h on ice. Eyecups were further cryopreserved in a 1:1 mixture [optimal cutting temperature medium (OCT):30% sucrose] overnight at 4°C. Cryopreserved eyecups were frozen in OCT. 5 µm cryosections were cut on a Medical Equipment Source 1000+ cryostat, mounted onto Superfrost Plus slides (VWR, 48311-701), and stored for less than 48 h at −80°C. For immunostaining, slides were warmed to room temperature prior to antigen retrieval in a 1× antigen retrieval solution (VWR, 103780-314) for 5 min at 80°C. Slides were equilibrated to room temperature, rinsed with 1×PBS and incubated in an antibody blocking solution [10% normal goat serum (NGS)+0.1% Triton X-100 in 1×PBS] for 1 h at room temperature. Sections were incubated in 1-2 µg of primary antibodies diluted another blocking solution (2% NGS+0.1% Triton X-100 in 1×PBS) at room temperature for 1 h. Sections were washed with PBS-T (0.1% Tween-20 in 1×PBS) four times for 5 min each prior to incubation with secondary antibodies diluted 1:500 in the same antibody blocking solution for 1 h at room temperature. Sections were washed in PBS-T and counterstained with 0.2 μg/ml 4′,6-diamidino-2-phenylindole (DAPI; Thermo Fisher Scientific, 62248) diluted in 1×PBS at room temperature for 20 min. Sections mounted with ProLong Glass Antifade Mountant (Thermo Fisher Scientific, P36980).

For immunofluorescence labeling of whole retinas for SIM, mouse eyes were enucleated, and corneas were punctured and fixed in a 4% PFA+Ames' medium (Sigma-Aldrich, A1420) fix solution for 15 min at room temperature. After the initial fixation, retinas were removed, bisected and cut into trapezoid segments. Retinal segments were fixed in 4% PFA+Ames' medium for an additional 45 min at room temperature for a total 1 h fixation. Retinas used for anti-Sec61β ER immunolabeling were lightly fixed for only 5 min total and stained as whole retinas. Retinas were quenched in 100 mM glycine in 1× PBS for 30 min at 4°C and then incubated in SUPER block buffer [15% NGS, 5% bovine serum albumin (BSA; Sigma-Aldrich, B6917)]+0.5% BSA-c (Aurion, VWR, 25557)+2% fish skin gelatin (Sigma-Aldrich, G7041)+0.05% saponin (Thermo Fisher Scientific, A1882022)+1× protease inhibitor cocktail (GenDepot, P3100-005), in half dram vials (Electron Microscopy Sciences, 72630-05) for 3 h at 4°C. Retinas were incubated with 5 µg (1:200 dilution) of primary antibodies that were spiked into the block buffer for 3 full days, at 4°C with gentle agitation. A second dose of either anti-Rho or anti-Sec61β antibodies (1:200 dilution) was added on the second day of primary antibody incubation to improve labeling. Retinas were washed six times with 2% NGS in Ames' medium for 10 min each on ice prior to incubate with 4 µg secondary antibodies (1:500 dilution) in 2% NGS in Ames+1× protease inhibitor cocktail for 12-16 h (overnight) at 4°C. Retinas were washed six times with 2% NGS in Ames' medium for 5 min each on ice and post-fixed in 2% PFA in 1×PBS for 30 min at room temperature with gentle agitation. Post-fixed retinas were then dehydrated with the following steps of pure ethanol diluted in water: 50%, 70%, 90%, 100%, 100%. Each ethanol step was performed at room temperature for 15 min with mild agitation. Following dehydration, retinas were embedded in Ultra Bed Low Viscosity Epoxy resin (Electron Microscopy Sciences, 14310) using the following steps (all room temperature with gentle agitation): 1:3 resin to 100% ethanol for 2 h; 1:1 resin to 100% ethanol for 2 h; 3:1 resin to 100% for ∼16 h (or overnight); two steps of full resin (no ethanol) 2 h each. Embedded retinas were then mounted in molds and cured for 24 h at 65°C. Resin blocks were trimmed and sectioned using glass knives on a Leica UCT Ultramicrotome to obtain 1-2 µm sections which were mounted on #1.5 glass coverslips with ProLong Glass Antifade Mountant.

### RNAScope

Frozen 5 µm retinal sections were collected on Superfrost Plus slides as described above. Sections were dried for 1 h at −20°C and stored overnight at −80°C. The RNAScope Multiplex Fluorescent Reagent Kit v2 [Advanced Cell Diagnostics (ACD), 323110] was used for RNA detection, as follows. Sections were postfixed with 4% PFA in 1×PBS for 5 min at room temperature and then dehydrated in ethanol steps (50%, 70%, 100%, 100%) for 5 min each step. Sections were dried and treated with hydrogen peroxide for 10 min at room temperature. Sections were then incubated in boiling Co-Detection Target Retrieval (ACD, 322000) for 2 min and then rinsed in water before incubation with 1 µg (1:200 dilution) anti-centrin primary antibody (Millipore Sigma, 04-1624) overnight at 4°C. The following day, sections were fixed in a 10% neutral buffered formalin solution for 30 min at room temperature, washed and incubated with Protease III (ACD, 322381) for 30 min in a 40°C hybridization oven. The RNA probes for *POLR2A*/positive control (ACD, 320881), *dapB*/negative control (ACD, 320871) and *Elfn1*/Mm-Efln1 (ACD, 449661) were added to sections for a 2 h incubation at 40°C. Amplification steps were then subsequently performed as per manufacturer instructions. Sections were then incubated with the HRP-C1 reagent (ACD, 323110) followed by the TSA vivid dye [ACD, 323271, 1:25,000 diluted in TSA buffer (ACD, 322809)] for 30 min at 40°C for probe visualization. Finally, sections were incubated with secondary antibody [F(ab′)2-goat anti-mouse Alexa 647, diluted 1:500] for 1 h at room temperature. Sections were counterstained with DAPI for 30 s and mounted with ProLong Glass Antifade Mountant. Slides were imaged on a Nikon N-SIM E microscope system (see below), and *z*-projections (step size, 0.2 μm) were obtained for reconstruction. RNA probe SIM channels (488 nm) were processed for additional 3D deconvolution. Identical acquisition settings were used for each imaging field.

### Fluorescence microscopy

Confocal microscopy was performed on either a Nikon C2 inverted confocal microscope, a Nikon AX inverted confocal microscope or a Nikon CrestV3 spinning disk microscope. Quantitative confocal imaging was performed on the C2 and AX systems using Plan fluor 40×/1.30 NA (C2), Plan flour 40×/1.30 DIC H N2 (AX) or Plan Apo λ 100×/1.45 NA oil immersion objectives. 405 nm, 488 nm, 561 nm and 640 nm laser lines were used on both systems. The CrestV3 spinning disk system was equipped with a Hamamatsu Fusion Gen III sCMOS camera, and a Plan Apo λD 60×/1.42 NA oil objective was used with Lumencor Celesta 405 nm, 477 nm, 546 nm and 638 nm laser excitation. For all confocal imaging, identical acquisition settings were used between age-matched WT control and mutant sections, which were always mounted and immunolabeled on the same slide. Confocal images were acquired using Nikon NIS-Elements software and processed and analyzed using Fiji/ImageJ (Schindelin et al., 2009). SIM imaging was performed at room temperature as described in Haggerty et al. (2024) using a Nikon N-SIM E microscope system equipped with a Hamamatsu Orca-Flash 4.0 camera and a SR HP Apochromat TIRF 100×/1.49 NA oil immersion objective. *Z*-projections were obtained with 0.2 µm *z*-steps (five to ten steps per image). SIM images were reconstructed using the NIS-Elements software and, in some cases, were additionally processed in NIS-Elements for 3D deconvolution using Automatic deconvolution mode. SIM images were processed and analyzed using Fiji/ImageJ.

### AAV subretinal injections

AAV constructs were designed and purchased from VectorBuilder. All AAV constructs contain a mouse rod specific MOPS500 promoter (Flannery et al., 1997). Human *RHO* coding sequences were tagged with an in-frame C-terminal TagRFP-T or EGFP. All AAVs contained an internal ribosome entry site (IRES) with a complimentary fluorescent tag (EGFP or mCherry) for visualization of AAV infection. AAVs were produced and packaged in the WVU Biochemistry and Molecular Medicine Virology Core. Three adult WT mice were injected with each AAV. Subretinal injections were performed as in Sechrest et al. (2024): prior to subretinal injection, mouse eyes were dilated with Tropi-Phen drops (Pine Pharmaceuticals). Mice were anesthetized using ketamine (80 mg/kg) and xylazine (10 mg/kg) in sterile 1×PBS via intramuscular injection. Fluorescein dye was added (0.1% final concentration) to AAVs for visualization. A 25-gauge needle was used to puncture the edge of the cornea. Transcorneal subretinal injections were performed by inserting a 33-gauge blunt end needle attached to a 5 μl Hamilton syringe containing 1 μl AAV and injecting into the subretinal space. After injection, a Neomycin+Polymixin B Sulfates+Bacitracin Zinc ophthalmic ointment (Bausch & Lomb) was added to the eyes, and antisedan (Orion Corporation) was intraperitoneally injected to reverse anesthesia.

Twenty-one days post-injection, mice were euthanized, and the injected eyes were enucleated. The corneas were punctured immersed in 4% PFA in Ames' medium. Eyes were fixed for 15 min before the cornea, lens and optic nerve were removed. Eyecups were then embedded in 4% low-melt agarose (Lonza, 50080). 150 µm vibratome sections were collected on a PELCO EasiSlicer vibratome and screened for AAV infection. Sections were stained for SIM by first quenching in a 100 mM glycine solution and blocking with 10% normal goat serum+0.1% Triton X-100 in 1×PBS. 1-2 µg primary antibodies were spiked into the blocking solution, and sections were probed for 12-16 h at 4°C with mild agitation. Sections were washed before incubation with secondary antibodies (diluted 1:500 in 1×PBS) for 2 h at room temperature. Sections were washed and post-fixed with 1% PFA prior to either sucrose cryopreservation and cryosectioning or ethanol dehydration and resin embedding as described above.

### Histology

For Hematoxylin and Eosin (H&E) staining and analysis, WT mice at P30 (*N*=3) and *WT-RFP/+* mice at P30 and P180 (*N*=3, each age) were euthanized, and the eyes were enucleated and immediately incubated into Excalibur's alcoholic Z-fix (Excalibur Pathology). Fixed eyes were sent to Excalibur Pathology (Norman, OK, USA) for paraffin sectioning and H&E staining. The H&E sections were imaged on a brightfield MIF Olympus Slide Scanner. Three H&E retinal sections through the optic disk were used for each of the mice included in the analysis. Photoreceptor nuclei in the ONL were counted in a masked analysis from an 80-µm-wide central retina region located 500 µm from the optic disk for each section.

### TEM

Mouse eyes were enucleated, the anterior segments were removed, and the eyecups were immersion fixed in ice-cold fixative (4% PFA+2.5% glutaraldehyde diluted in 1×PBS, pH 7.4) for 12-16 h at 4°C with mild agitation. Retinas were dissected from the fixed eyecups and cut into four trapezoidal pieces. Retinas were rinsed with 100 mM cacodylate buffer (pH 7.4) three times and post fixed in 1% OsO4+1.5% K_4_[Fe(CN)_6_]×3H_2_O in 100 mM cacodylate buffer for 1 h at 4°C with mild agitation. Retinas were then washed three times in a wash solution (100 mM cacodylate buffer+50 mM Na-maleate, pH 5.0, in water) on ice, 5 min each step. Then, retinas were incubated in 2% uranyl acetate (UA) in 50 mM Na-maleate for 3 h at 4°C with mild agitation. Retinas were washed with 50 mM Na-maleate three times and water three times on ice for 5 min each step before dehydration with increasing concentrations of ethanol (50%, 70%, 90%, 100%, 100%) and two 100% acetone steps at room temperature for 15 min each step. Dehydrated retinas were resin embedded in increasing concentrations of Eponate 12 resin (Ted Pella, 18010) in room temperature/mild agitation stages, as follows: 1:1 resin to acetone overnight; 3:1 resin to acetone 2 h; two full resin steps (no acetone), 2 h each. Embedded retinas were transferred to molds and cured at 65°C for 48 h. Ultrathin resin sections (70 nm) were cut on a Leica UCT ultramicrotome using a Diatome Ultra 45° diamond knife and collected on copper grids (Electron Microscopy Sciences, G100-Cu). Copper grids were post stained with a 1.2% UA solution and a 3% lead citrate solution (Electron Microscopy Sciences, 22410) for 4 min each. Grids were imaged on either a Joel JEM 1010 transmission electron microscope or a Joel 1400 transmission electron microscope.

### Immuno-EM

Immuno-EM methods were adapted from the literature (Puthussery and Fletcher, 2004). Mouse eyes were enucleated, the anterior segments were removed, and the eyecups were immersion fixed in ice-cold fixative (4% PFA, 0.1% glutaraldehyde, 1 mM CaCl_2_ in 1×PBS) for 30 min with mild agitation. Retinas were them placed in a secondary fix (4% PFA, 1 mM CaCl_2_ in 1×PBS) with mild agitation at 4°C. Fixative was replaced with blocking buffer [10% NGS, 1% BSA in 1×PBS (pH 7.4)], and retinas were blocked at 4°C for 2 h with mild agitation. Primary antibody was diluted in antibody solution [3% NGS, 1% BSA in 1×PBS (pH 7.4)] for primary staining of retinas for 4 days at 4°C with mild agitation. Sections underwent a series of washes with 1×PBS before secondary staining (1:200 dilution in antibody solution) for 2 h at room temperature with mild agitation. Retinas were washed with 1× PBS before undergoing secondary antibody visualization with the VectaStain ABC Kit (Vector Laboratories, PK-6100) following the manufacturer’s protocol. Retinas were then washed with 1×PBS followed by washing with 0.05 M Tris-HCl (pH 7.6). Retinas were then incubated in a DAB solution (0.05% DAB in 0.05 M Tris-HCl) and incubated at room temperature for 15 min with mild agitation before a second DAB step (0.05% DAB in 0.01% H_2_O_2_) following the same incubation conditions. Retinas were washed for 5 min in 0.05 M Tris-HCl (pH 7.6) to stop the reaction. Retinas were washed with water followed by a wash in 0.1 M cacodylate and then post-fixed (2.5% glutaraldehyde in cacodylate buffer) for 2 h at 4°C with mild agitation. Retinas were washed with water before undergoing silver intensification (2.6% hexamethylenetetramine, 0.2% AgNO_3_, 0.2% sodium borate) for 10 min at 60°C followed by gold toning (0.05% gold chloride in water) for 10 min at 4°C, with water washes between incubations. Retinas were washed in water before the addition of 0.05% oxalic acid diluted in water for 2 min. Retinas were then incubated for 1 h in fresh 1% sodium thiosulfate in water at room temperature. Retinas then underwent a secondary post-fix (0.05% OsO_4_ in cacodylate buffer) before ethanol dehydration and resin embedding as described above.

### TMT-MS

Whole mouse retinas were dissected for TMT-MS in sterile 1×PBS, and the ciliary bodies were removed. Retinas from both eyes of each mouse were combined and flash frozen on dry ice. For P30 analysis, three WT and three *P23H-RFP/+* males were used. For P90 analysis, three WT female and four *P23H-RFP/+* female mice were used. Frozen retinal samples were sent to IDEA National Resource for Quantitative Proteomics (Little Rock, AR, USA) for lysis, trypsin digestion, tandem mass tag labeling and Orbitrap Eclipse MS acquisition. Database analysis, quality control, normalization, fold-change calculations and statistical testing (described below) were performed by IDEA. All acquired TMT-MS data are available in Table S1, and Scaffold files for evaluating protein database search results are openly available via Mendeley Data at doi:10.17632/w4d49nkm5n.2.

### Western blotting

Three WT and three *P23H-RFP/+* mice were used for P30 and P90 western blot analyses. Dissected retinas were flash frozen on dry ice for at least 10 min, resuspended in 100 µl of 1% Triton X-100+1× protease inhibitor (Thermo Fisher Scientific, A32955) in 1×PBS and lysed by sonication. Lysed samples were cleared with centrifugation, and 11 µl of each sample was combined with 11 µl urea sample buffer [6 M urea+140 mM SDS+0.03% Bromophenol Blue+360 mM β-mercaptoethanol in 0.125 M Tris-HCl (pH 6.8)] and heated for 5 min at 95°C. Samples were loaded into a Novex Tris-glycine mini 4-12% gel (Thermo Fisher Scientific, XP04120) for SDS-PAGE. Gels were transferred onto Immobilon-FL Transfer Membrane polyvinylidene difluoride (pore size, 0.45 μm) (LI-COR, 92760001) in Tris-Glycine Transfer Buffer (Bio-Rad, 1610771). Blots were blocked with Intercept Blocking Buffer (LI-COR, 927–6000) for 1 h and then washed three times with PBS-T for 5 min each. Primary antibodies at 1:500 to 1:20,000 dilutions in PBS-T were added to the blots for 2 h probing at room temperature. Blots were washed and secondary antibodies (diluted 1:50,000 in PBS-T) were added for 1 h probing at room temperature. Blots were imaged on an Amersham Typhoon scanner (GE Healthcare).

### Deglycosylation assay

Deglycosylation assays were performed as in Haggerty et al. (2024): dissected retinas were flash frozen at −80°C for 10 min and then lysed in 100 µl RIPA buffer (Alfa Aesar, J63306)+protease inhibitor cocktail (GenDepot, P3100-001). Samples were cleared with centrifugation, and the cleared supernatant was used for the assay. Lysate was mixed with deglycosylation buffer containing PNGase F (New England Biolabs, P6044) and incubated for 10 min at 37°C. Protein deglycosylation mix II (New England Biolabs, P6044S) was added to the treated tubes, and buffer only was added to the control tubes. All samples were then incubated for 1 h at 37°C. Samples were cooled on ice for up to 10 min, mixed with urea sample buffer for a 1:1 mixture and loaded onto IVGN Novex WW 10-20% Tris-Glycine gels (Fisher Scientific, 89238-778) for western blotting, as described above.

### Image processing and analysis

For confocal image puncta analyses (Fig. 5E,F, Fig. 7J; Fig. S4C,D), channels of interest were cropped to a width of 80 µm and coded for a masked analysis. For Dmd and Bsn, integrated densities of ten individual puncta were measured for each of four images per mouse included in the analysis (40 puncta per mouse in total). An equal number of mean background measurements were taken for each image. The average mean background was multiplied by the puncta areas, and these values was used for background subtraction. For the ELFN1 OPL particle analysis, the OPL was selected based on DAPI staining, and thresholding was used to collect the foreground ELFN1 puncta signal and background for each image. Thresholding values were identical for all images and conditions. For ELFN1 and mGluR6 ONL versus OPL intensity measurements (Fig. 6A-D; Fig. S5D), mean intensity values were measured from ONL and OPL regions, which were selected based on the DAPI channel. For the RFP puncta analysis, channels were selected as before, and puncta were preselected and coded based on their association (RFP^+^) or lack of association (RFP^−^) with RFP signal for masked integrated density measurements. RNAScope puncta were counted from SIM *z*-projections by selecting IS, ONL and OPL regions based on the centrin immunostaining and DAPI channels. In SIM images, the dONL was distinguished in images focused on the IS region from the proximal ONL (pONL) in images focused on the OPL. Analyze Particles in FIJI/ImageJ was used for puncta counting in each region, and the same thresholding values were used for all images. For TEM, images that contained a clear ribbon in the front-view, rod-like orientation were selected for a masked analysis based on the parameters described in Kesharwani et al. (2021). The ribbon height was manually measured from the anchoring site to the ribbon tip, and the synaptic vesicles were manually counted and considered ribbon associated if they overlapped or had clear tethers to the ribbon. For western blotting, the intensities of the bands from the blot scan images were measured using Fiji/ImageJ. Background measurements were also taken from the same area as the measured bands for background correction.

### Experimental design and statistical analysis

Specific experimental design details, such as number of mice and cells examined, are included in the Results section or figure legends. Sample size was determined based on a power analysis. All confocal analyses were performed with matching retinal sections between experimental and WT control conditions. Experimental datasets were directly compared to the matching WT data, and thus all data were normalized for aggregation so that WT mean values=1. To statistically compare the aggregated data, standard deviations were propagated to determine the relative error, and the propagated standard deviations are represented as error bars in each of the aggregated data graphs. For TMT-MS data, volcano plots were generated using VolcaNoseR (Goedhart and Luijsterburg, 2020), and gProfiler was used to classify proteins based on the Gene Ontology Cell Compartment (GO-CC) terms: ‘photoreceptor outer segment’, ‘synapse’, ‘photoreceptor inner segment’ and ‘photoreceptor connecting cilium’. TMT-MS data were statistically compared with a differential abundance analysis and moderated *t*-tests to account for protein variability, distribution and abundance. For each comparison, *P*-values and false discovery rate-adjusted *P*-values were calculated. All graphs were generated using GraphPad Prism, and all statistical testing was performed in either GraphPad Prism or GraphPad Quickcalcs. WT data were normalized to a value of 1, and mutant data were then normalized to this value. In all graphs, bars represent aggregate mean aggregate values and error bars indicate standard deviations after error propagation unless stated otherwise. Unless specified otherwise, significance was determined using unpaired two-tailed *t*-tests with error bars representing s.e.m. All original data corresponding to the graphs are openly available via Mendeley Data at doi:10.17632/w4d49nkm5n.2.

## Supplementary Material

10.1242/dmm.052256_sup1 Supplementary information

Table S1. TMT-MS results.
