# Supplementary material for: P23H rhodopsin accumulation causes transient disruptions to synaptic protein levels in rod photoreceptors in a model of retinitis pigmentosa
Source: Dis Model Mech. 2025 Jun 23;18(6):dmm052256. doi: 10.1242/dmm.052256 (PMC12233068; doi:10.1242/dmm.052256)
Supplement: Supplementary information [file dmm-18-052256-s1.pdf]

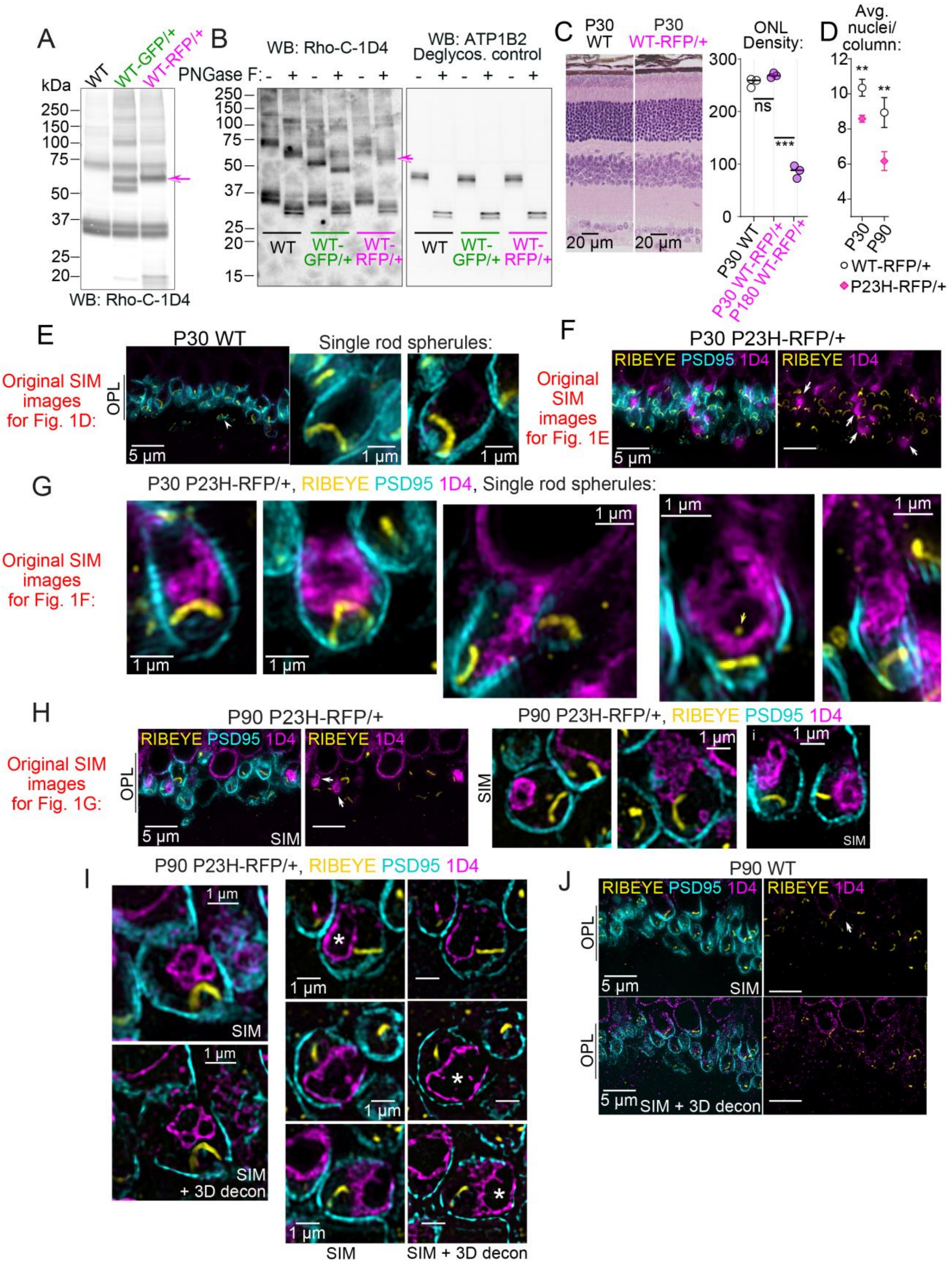

A
WT
WT-GFP/+
WT-RFP/+
kDa
250
150
100
75
50
37
25
20
WB: Rho-C-1D4
B
WB: Rho-C-1D4
PNGase F: - + - + - +
WT
WT-GFP/+
WT-RFP/+
WB: ATP1B2
Deglycos. control
C
P30 WT
P30 WT-RFP/+
20 μm
ONL Density:
ns
***
P30 WT
P30 WT-RFP/+
P180 WT-RFP/+
D
Avg. nuclei/ column:
P30
P90
WT-RFP/+
P23H-RFP/+
E
P30 WT
Single rod spherules:
Original SIM images for Fig. 1D:
OPL
5 μm
1 μm
F
P30 P23H-RFP/+
RIBEYE PSD95 1D4
RIBEYE 1D4
Original SIM images for Fig. 1E
G
P30 P23H-RFP/+, RIBEYE PSD95 1D4, Single rod spherules:
Original SIM images for Fig. 1F:
H
P90 P23H-RFP/+
Original SIM images for Fig. 1G:
SIM
P90 P23H-RFP/+, RIBEYE PSD95 1D4
I
P90 P23H-RFP/+, RIBEYE PSD95 1D4
SIM + 3D decon
J
P90 WT

Fig. S1. Supplemental data related to Fig. 1. (A) Western blots of WT (age P77), *Rho-GFP-1D4/+* (abb: *WT-GFP/+*, age P61), and *WT-RFP/+* (age P58) retinal lysates. In *WT-GFP/+* and *WT-RFP/+* lanes, 60-65 kDa Rho-C- 1D4-positive bands corresponding to the Rho-GFP/RFP fusions are larger than the ~35 kDa endogenous monomer Rho protein bands that is present in all lanes. There is an additional, smaller band in the *WT-GFP/+* lane that cannot be identified. Magenta arrow = the band corresponding to WT-hRho-RFP. (B) Western blots of WT (age P70), *WT-GFP/+* (age P67) and *WT-RFP/+* (age P58) retinal lysates after treatment with PNGase F or buffer only. Rho protein deglycosylation shifts were evident with Rho-C-1D4 immunolabeling, including shifts in the WT-hRho-RFP band (magenta arrow). Na, K ATPase beta 2 (ATP1B2) deglycosylation serves as the control (“Deglycos. control”). (C) H&E stained central retina example sections from P30 WT (N=3 mice) and P30 *WT-RFP/+* (N=3 mice) retinas. ONL density values (# of photoreceptor nuclei per 80 µm central retina region) are plotted for P30 WT, P30 *WT-RFP/+* and P180 *WT-RFP/+* (N=3 mice). Bars = mean values. (D) DAPI+ nuclei per ONL column values from confocal images like in Fig. 1 A-B were plotted for *WT-RFP/+* and *P23H-RFP/+* mice at ages P30 and P90 (N=3 mice, each group at each age). (E-H) SIM images without 3D deconvolution for the denoted main figure panels. (I) P90 P23H-RFP/+ single spherule examples. White arrows = 1D4 puncta localized in the *P23H-RFP/+* OPL. White asterisks = gaps in the accumulated 1D4 fluorescence in P23H-RFP/+ spherules. (J) SIM images of WT OPL at age P90. 1D4 signal was not observed in the OPL.

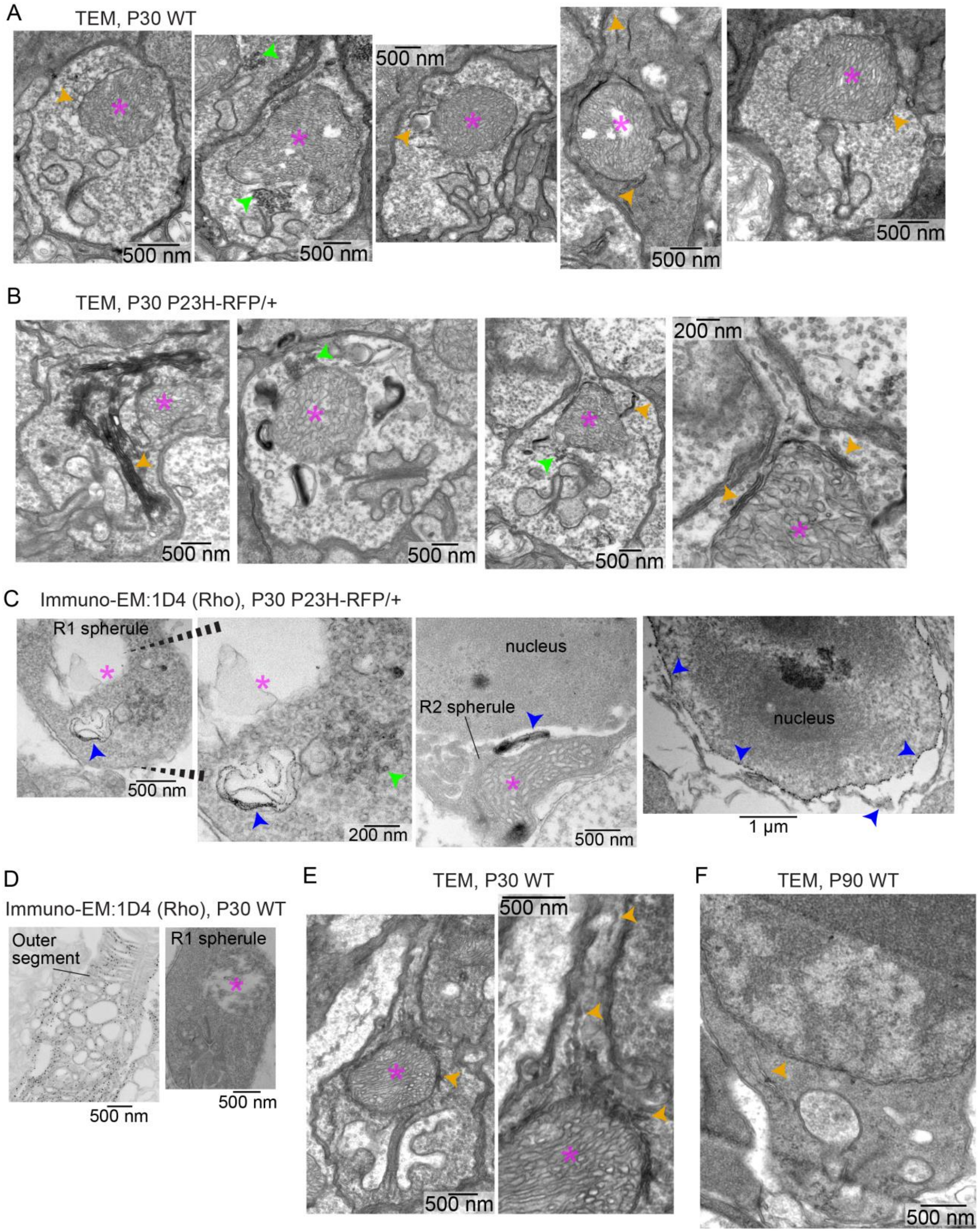

Disease Models & Mechanisms • Supplementary information

Fig. S2. Supplemental data related to Fig. 2. (A) TEM images of P30 WT spherules. Magenta asterisks = mitochondria, green arrowheads = endocytosed vesicles). ER-like membranes (orange arrowhead) are located near the mitochondria. (B) TEM images of P30 *P23H-RFP/+* rod spherules annotated as in (A). Denser ER membranes are observed in these mutant spherules (orange arrowheads). (C) Immuno-EM images of sections stained for Rho in P30 P23H-RFP/+ retinas. Blue arrowheads = immunopositive signal in ER-like structures. (D) P30 WT Immuno-EM images: Rho signal can be seen in the outer segment (left) but not the spherule example (right). (E) A P30 WT R1-type rod spherule. ER (orange arrowheads) is observed in the axon and surrounding the spherule mitochondrion. (F) TEM image of an R2 spherule in a P90 WT retina depicting ER-like membranes extending from around the nucleus and into the spherule of the cytoplasm.

A

TMT-MS values and P-values corresponding to Fig. 4 C and E and other synaptic proteins:

P30									
	WT 1	WT 2	WT 3	P23H-RFP/+ 1	P23H-RFP/+ 2	P23H-RFP/+ 3	P-Value	Adjusted P-Value	# of unique peptides
Rho	27.3313	27.4507	27.1987	25.1108	25.4229	25.0575	1E-06	0.0003736	5
Prph2	26.9136	26.8207	26.7683	26.1727	26.3159	25.9264	0.00042	0.0158367	12
Pdc	26.7152	26.6583	26.7337	26.8454	26.8191	26.6886	0.16061	0.3871834	15
Reep6	23.3942	23.3525	23.3365	23.6956	23.6306	23.4483	0.01338	0.1043769	7
Tulp1	24.2303	24.2842	24.1951	24.0335	23.9133	23.8705	0.00118	0.0267525	19
Elfn1	19.2768	19.4406	19.274	18.9248	18.9967	18.7365	0.00143	0.0296644	5
Grm6	19.2799	19.4496	19.2958	19.3355	18.9263	19.1798	0.12295	0.3371009	6
Dag1	20.4382	20.3864	20.4144	20.3334	20.5155	20.5655	0.39832	0.626117	6
Dmd	21.7629	21.7804	21.7799	21.5833	21.6785	21.5857	0.00552	0.062718	24
Egflam	20.1292	20.1208	20.0968	20.0596	19.8303	19.9349	0.02773	0.1534834	6
Erc1	20.6803	20.7683	20.7233	20.7979	20.8326	20.7826	0.07003	0.2470412	10
Rims2	19.6159	19.6906	19.7093	19.4495	19.5648	19.4101	0.00861	0.080247	11
Bsn	22.7665	22.7935	22.7791	22.7007	22.7238	22.603	0.04501	0.1968775	46
Guca1b	21.1121	20.9938	21.0996	20.3738	19.4637	20.0672	0.00325	0.046721	
Cabp4	15.9897	16.2738	16.1386	16.1106	15.7326	16.0776	0.23088	0.4705738	
Dlg4	23.8998	23.9194	23.9272	23.8036	23.7849	23.744	0.00483	0.0573932	19
Gpr179	20.7096	20.6348	20.679	20.4828	20.5601	20.4205	0.0065	0.069647	18
Cacna1f	no peptides detected								
Cacna2d4	19.7171	19.7003	19.7183	19.7419	19.7517	19.6337	0.9494	0.9806384	5
Dnm1	26.2076	26.2334	26.2332	26.2368	26.1954	26.1461	0.40635	0.6336635	28
Dnm3	24.5546	24.5758	24.5805	24.5779	24.5129	24.5888	0.77335	0.8896834	27
Cplx3	24.3634	24.4909	24.3647	24.3026	24.3708	24.1685	0.10007	0.2998617	10
Syt1	27.0361	27.0441	27.0413	27.0081	27.0261	26.9721	0.26035	0.4994329	13
Syt7	18.7408	18.7239	18.7359	18.8315	18.7287	18.7276	0.49166	0.6997498	4

P90										
	WT 1	WT 2	WT 3	P23H-RFP/+ 1	P23H-RFP/+ 2	P23H-RFP/+ 3	P23H-RFP/+ 4	P-Value	Adjusted P-Value	# of unique peptides
Rho	27.7452	27.8304	27.8807	26.4843	26.364	26.5647	26.1675	8E-07	0.00026	6
Prph2	26.1818	26.1831	26.2894	25.7262	25.6027	25.6892	25.4338	7E-05	0.00494	11
Pdc	26.0464	26.1301	26.0644	26.5701	26.3919	26.5398	26.397	0.00024	0.00865	16
Reep6	23.0354	23.0382	23.1073	23.3797	23.3977	23.325	23.404	0.00011	0.006	7
Tulp1	23.8988	23.9181	24.0056	23.5885	23.7007	23.7284	23.7893	0.00406	0.04401	21
Elfn1	19.9656	20.0142	19.9827	19.7595	19.644	19.8362	19.8305	0.00542	0.05191	5
Grm6	20.4182	20.0391	20.3554	19.7879	19.7522	19.7038	19.9635	0.00233	0.03353	9
Dag1	19.6672	19.6093	19.5415	19.7413	19.839	19.8271	19.8971	0.00385	0.04268	7
Dmd	20.7033	20.6781	20.7474	20.626	20.7364	20.5375	20.7524	0.47486	0.66894	19
Egflam	18.2079	18.5186	18.4116	18.2117	17.946	18.1471	17.9515	0.01321	0.08531	6
Erc1	18.928	18.9107	18.9647	19.1222	19.1487	19.143	19.2928	0.00219	0.03234	7
Rims2	16.2425	16.3166	16.269	15.9725	15.8789	16.0512	15.821	0.00072	0.01592	4
Bsn	21.8302	21.8943	22.0146	21.8401	21.9614	21.9014	22.1543	0.55742	0.73452	52
Guca1b	20.5326	20.4376	20.4076	19.9833	19.5186	19.6829	19.7516	0.00016	0.00717	
Cabp4	19.1312	19.2365	19.0694	18.7708	18.6666	18.7475	18.8177	0.00018	0.00758	
Dlg4	23.7351	23.7495	23.7803	23.6368	23.6133	23.5391	23.6584	0.01324	0.08531	17
Gpr179	20.1679	20.1335	20.2558	20.015	20.0835	20.0025	20.1563	0.06159	0.20263	15
Cacna1f	13.5825	13.5835	13.3883	13.1042	13.3277	13.4593	13.2791	0.04064	0.15959	0
Cacna2d4	19.0163	19.0197	19.0496	19.0631	18.9873	19.0146	18.9102	0.48644	0.67556	7
Dnm1	23.8637	23.8824	23.9203	23.8982	23.9636	23.9532	23.8513	0.55397	0.73173	28
Dnm3	23.9432	23.9473	23.9314	23.9633	23.9359	23.9815	23.7351	0.59312	0.76012	23
Cplx3	24.8706	24.8483	24.8732	24.7183	24.8055	24.8908	25.031	0.97215	0.98759	10
Syt1	26.9321	26.9107	26.8771	26.9125	26.8963	26.9784	26.7683	0.76155	0.87062	17
Syt7	17.5321	17.4769	17.5615	17.5261	17.5463	17.5066	17.6104	0.60957	0.77195	4

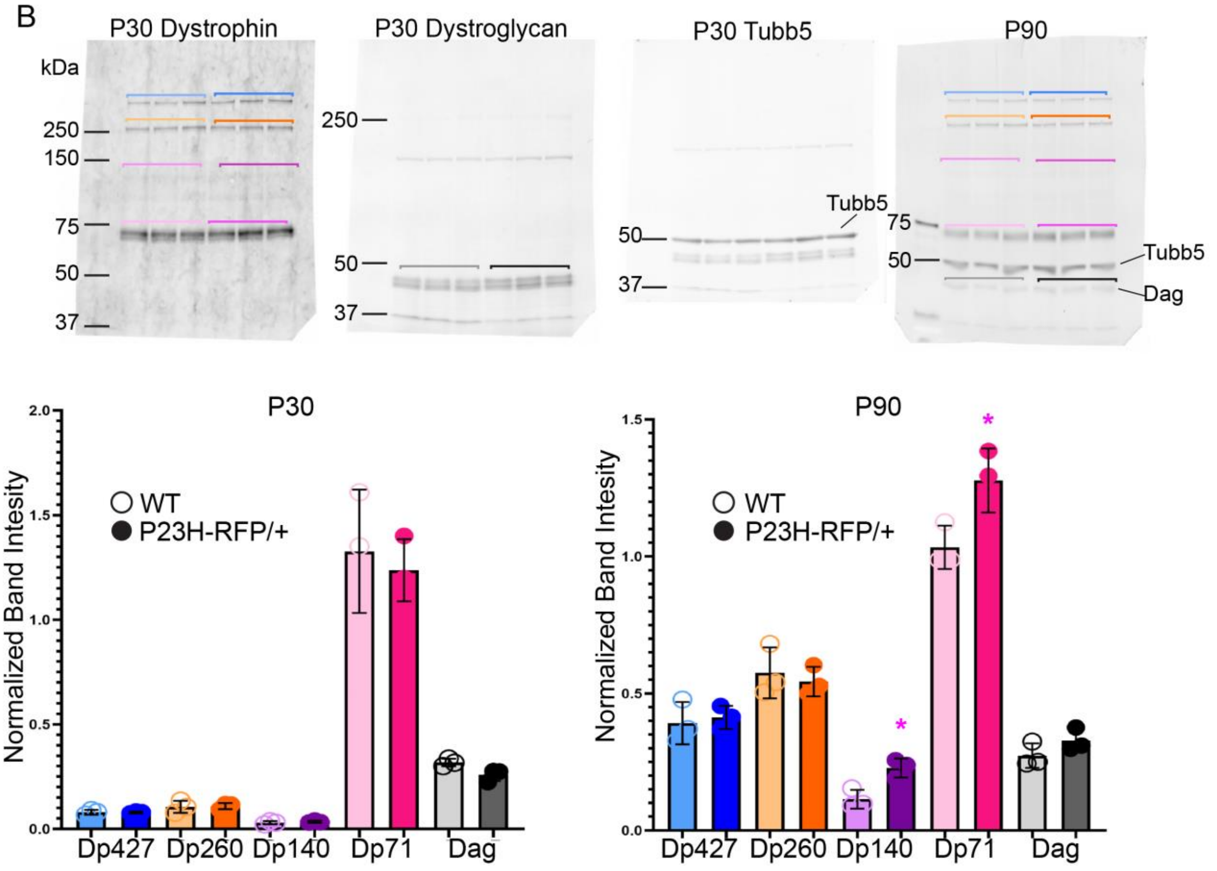

Fig. S3. Supplemental data related to Fig. 4. (A) SIM image of the OPL of a P30 WT retina. The rod synaptic protein ELFN1 (cyan) is typically located beneath the synaptic ribbon (yellow). Gaps in a single ELFN1 fluorescent punctum suggest invaginating post-

synaptic neurites (dotted white lines). (B) Table of normalized TMT-MS values for select proteins, including the proteins in Fig. 4 B and D. Dag1 = Dystroglycan. (C) Western blot analysis for Dystrophin isoforms in WT and *P23H-RFP/*+ whole retinas at ages P30 and P90. Each lane represents a retinal lysate sample from a separate mouse (N=3). Colored brackets on the blots correspond to the colored bars in the graphs for densitometry intensity quantification. All intensities are plotted as normalized to corresponding tubulin band intensities (Tubb5). Asterisks indicate significance (P = <0.05) based on an unpaired t-test.

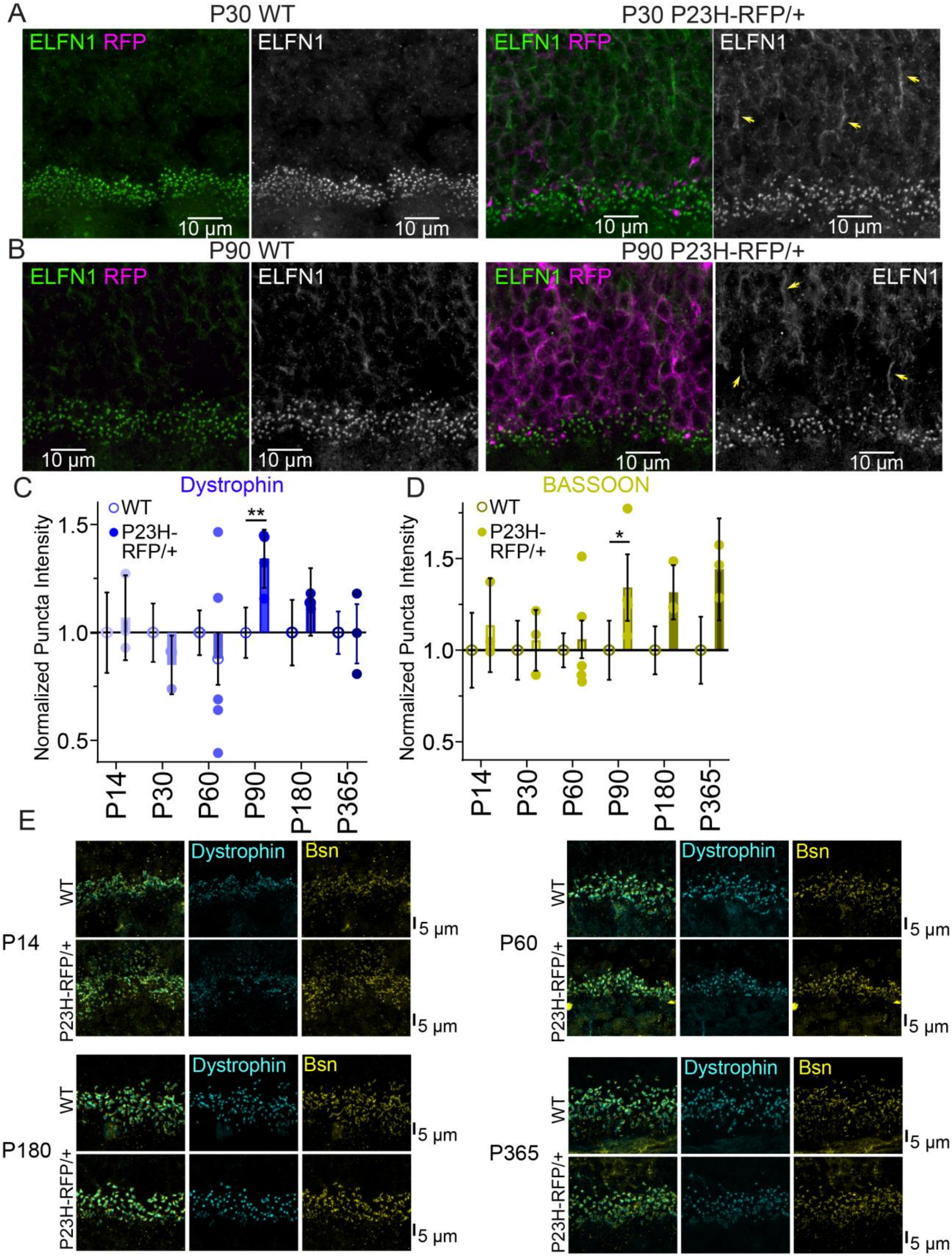

Disease Models & Mechanisms • Supplementary information

Fig. S4. Supplemental data related to Fig. 5. (A, B) Confocal z-projections of replicate WT (left) and P23H-RFP/+ (right) retinas immunolabeled for ELFN1 (green) at ages (A) P30 or (B) P90. ELFN1 and RFP (magenta) levels were matched between WT and *P23H-RFP/+* images. Yellow arrows = strings of ELFN1 in the *P23H-RFP/+* ONL. For (A) and (B), these are the same samples and images used in Fig. 5C,D. (C, D) Graphs of normalized Dystrophin and BASSOON intensity values corresponding to the data in Fig. 5 G. (E) Example confocal z-projections from WT and *P23H-RFP/+* retinal cryosections at P14, P60, P180 and P365 focused on the OPL with Dystrophin (cyan) and BASSOON (Bsn, yellow) immunolabeling.

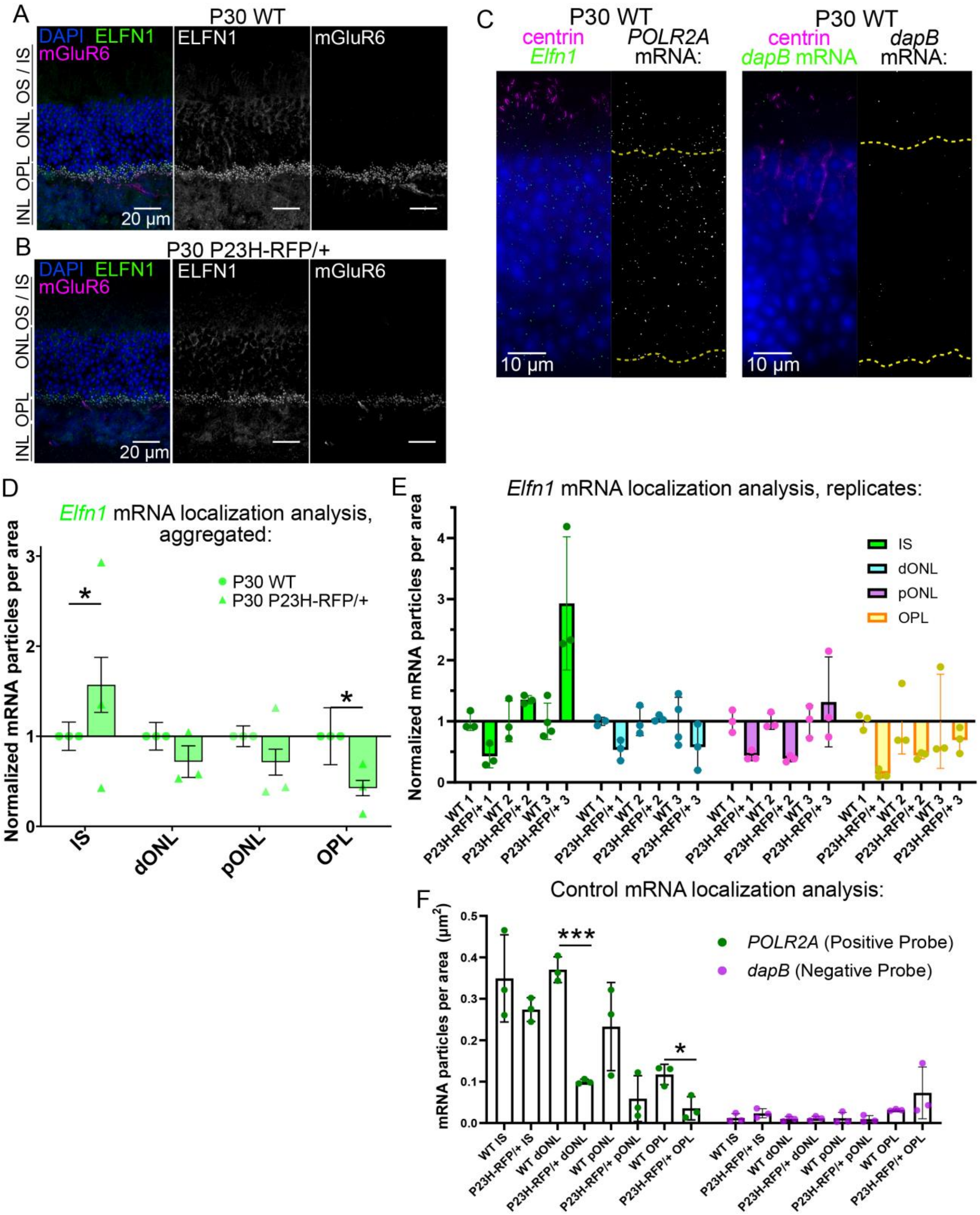

Disease Models & Mechanisms • Supplementary information

Fig. S5. Supplemental data related to Fig. 6. (A, B) Confocal z-projection images of replicates for Fig. 6 A-B. (C) Example RNAscope SIM z-projections images for *POLR2A* (positive control) and *dapB* (negative control) mRNAs in P30 WT retina sections; additional images for Fig. 6E. (D) Graph *Elfn1* mRNA particles per area (μm^2) from RNAScope experiments. *Elfn1* counts are graphed for the IS, dONL, pONL, and OPL layers from P30 WT (circles) and P30 P23H-RFP/+ (triangles) retinas. Values are from replicate WT vs *P23H-RFP/+* comparisons and all data were normalized to WT mean = 1. (E) Graph of the de-aggregated, normalized Elfn1 mRNA particles per area data corresponding to Fig. S5D. (F) Graph of replicate, normalized mRNA particles per area values for the control RNAscope probes in Fig. 6E.

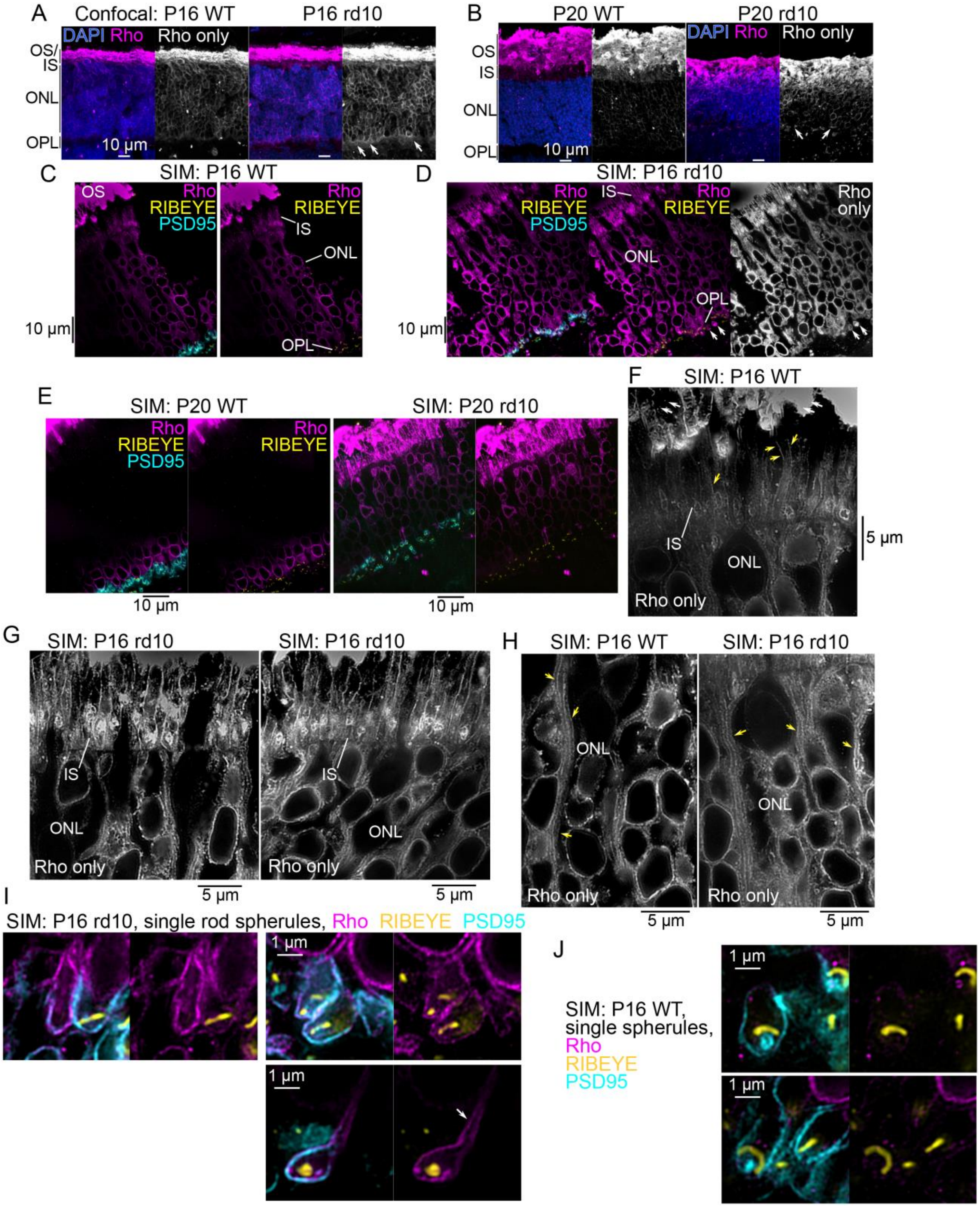

Disease Models & Mechanisms • Supplementary information

Fig. S6. Supplemental data related to Fig. 7. (A, B) Confocal z-projections of retinal cryosections from WT (N=3 mice) and rd10 mice at age P16 (N=5 mice) (A) or P20 (N=3 mice) (B) immunolabeled with the 4D2 Rho antibody (magenta) and counterstained with DAPI (blue). Mislocalized 4D2+ Rho signal is present in the rd10 OPLs (white arrows). (C) SIM images of a P16 WT retina immunolabeled for 4D2 (Rho, magenta), RIBEYE (yellow), and PSD95 (cyan). Rho fluorescence was detected throughout the OS, IS, and ONL but not in the OPL based on images with (left) and without (right) PSD95. (D) SIM image of a P16 rd10 retina labeled as in (C). Rho fluorescence was detected throughout the OS, IS, ONL, and also mislocalized in the OPL (white arrows). Settings and intensity levels were matched between (C) and (D). (E) SIM example images of P20 WT (left) and rd10 (right) retinas. 4D2+ Rho signal (magenta) is localized in WT rods at the bottom of the ONL near the spherules (cyan) and ribbons (yellow), but not in the OPL. (F) 4D2+ Rho fluorescence in a SIM image of a P16 WT retina. Rho is located along the IS plasma membrane (yellow arrows). (G) 4D2+ Rho fluorescence in SIM example images of P16 rd10 retinas demonstrates Rho localization at the IS plasma membrane and myoid region. (H) More 4D2+ Rho fluorescence in SIM images from P16 WT and rd10 retinas focused on the ONL. (I) SIM single spherule example images from P16 rd10 retinas. 4D2+ Rho (magenta) is colocalized with PSD95 (cyan) at the spherule plasma membrane and along rod axons (white arrows). (J) SIM super-resolution single spherule images from P16 WT retinas.

Table S1. TMT-MS results.

Available for download at
https://journals.biologists.com/dmm/article-lookup/doi/10.1242/dmm.052256#supplementary-data

Disease Models & Mechanisms • Supplementary information
